# 3-(3-Azabicyclo[2, 2, 1]heptan-2-yl)-1,2,4-oxadiazoles as Novel Potent DPP-4 Inhibitors to Treat T2DM

**DOI:** 10.3390/ph18050642

**Published:** 2025-04-28

**Authors:** Tatiana V. Zinevich, Ivan O. Maslov, Olga G. Kirichenko, Sergey V. Shorshnev, Maxim A. Gureev, Fedor M. Dolgushin, Yuri B. Porozov, Vladimir M. Trukhan

**Affiliations:** 1 Department of Bioorganic Chemistry, Faculty of Biology, Lomonosov Moscow State University, 119991 Moscow, Russia; zinevich.tatiana@gmail.com (T.V.Z.); drivanmaslov@gmail.com (I.O.M.); 2 LLC Institute of Mitoengineering MSU, 119899 Moscow, Russia; kirolga2001@mail.ru; 3 MIREA, Russian Technological University, Campus in Malaya Pirogovskaya Street, 119435 Moscow, Russia; gray20002006@yandex.ru; 4 Laboratory of Bio- and Chemoinformatics, HSE University, 16 Soyuza Pechatnikov, 190121 Saint-Petersburg, Russia; max_technik@mail.ru (M.A.G.); yuri.porozov@gmail.com (Y.B.P.); 5 Kurnakov Institute of General and Inorganic Chemistry RAS, 119071 Moscow, Russia; fmdolgushin@gmail.com; 6 Institute for Translational Medicine and Biotechnology, I. M. Sechenov First Moscow State Medical University, 119991 Moscow, Russia

**Keywords:** type 2 diabetes mellitus, DPP-4 inhibitors, molecular docking, structure–activity relationship, stereoisomerism, oxadiazoles

## Abstract

**Background**: Type 2 diabetes mellitus (T2DM) is a prevalent metabolic disease with global implications, necessitating effective management strategies. Dipeptidyl peptidase IV (DPP-4) inhibitors have shown promise as potent agents for T2DM treatment. **Methods**: This study combines chemical synthesis, molecular modelling, and inhibitory activity assays to characterise the structure–activity relationship of novel isomeric 1,2,4-oxadiazole-substituted derivatives of the 2-azabicyclo[2.2.1]heptane scaffold acylated with (*R*)-3-amino-4-(2,4,5-trifluorophenyl)butanoic acid. **Results**: In this article, we demonstrate the efficacy of new compounds as robust inhibitors of DPP-4. The attempts to further modify neogliptin (our lead compound described previously) resulted in a more potent DPP-4 inhibitor **9a** (IC_50_ = 4.3 nM), which did not mediate any substantial inhibition of DPP-8 and DPP-9. **Conclusions**: This study demonstrates that pseudo peptides incorporating (*R*)-3-amino-4-(2,4,5-trifluorophenyl)butanoic acid, a 2-aza-bicyclo[2.2.1]heptane moiety, and 1,2,4-oxadiazole substituents act as potent and selective DPP-4 inhibitors. By the stereochemical refinement of oxadiazole derivatives of neogliptin, we discovered compound **9a**, a strong candidate for further development in T2DM treatment.

## 1. Introduction

Type 2 diabetes mellitus (T2DM) is a complex chronic metabolic disease characterised by hyperglycaemia arising from both insulin resistance [1] and impaired insulin production [2]. T2DM affects millions of people worldwide and is associated with various complications (cardiovascular, nephro-, neuro-, and retinopathy, and foot ulcers) [3]. To effectively manage T2DM and its complications, early diagnosis, lifestyle modifications, and medical interventions are required [4]. Multiple antihyperglycemic treatment options are approved to provide personalised diabetes care, with ongoing clinical investigations exploring novel drugs [5].

**Figure 1:**
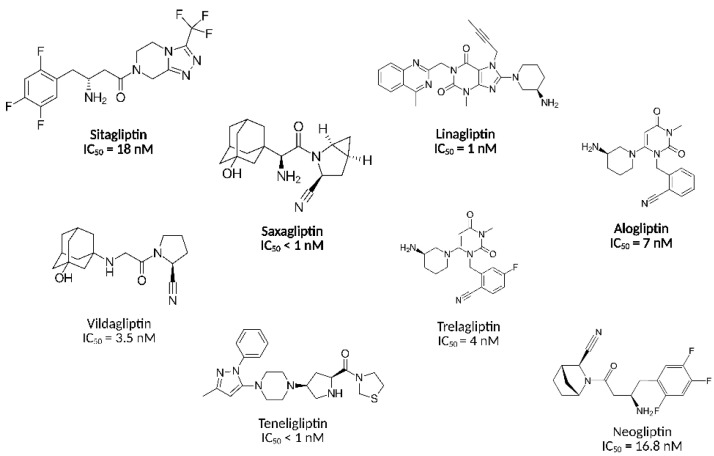
Structures and DPP-4 inhibitory activities of selected compounds. The U.S. Food and Drug Administration (FDA)-approved drugs are shown in bold.

Among those, dipeptidyl peptidase 4 (DPP-4) inhibitors, commonly called gliptins, have garnered considerable attention; these orally administered small-molecule agents prevent the fast enzymatic degradation of incretin hormones by inhibiting DPP-4. This therapeutic approach augments the levels of glucagon-like peptide-1 (GLP-1) and glucose-dependent insulinotropic polypeptide (GIP) that stimulate insulin synthesis and suppress glucagon secretion [6]. DPP-4 inhibitors have demonstrated their efficacy in reducing glycated haemoglobin (HbA1c) levels as monotherapy and in combination with other antidiabetic agents [5]. Notably, DPP-4 inhibitors are suitable for patients intolerant to metformin [7] and less likely to cause hypoglycaemia. In other words, their favourable safety profile makes DPP-4 inhibitors ideal for a diverse patient population [8]. Several structures with target-specific interaction with DPP-4 have received official approval from the U.S. Food and Drug Administration (FDA), including sitagliptin [9], saxagliptin [10], alogliptin [11], and linagliptin [12] (Figure 1). Although the FDA has not yet approved some other gliptins (e.g., vildagliptin, teneligliptin, trelagliptin), at least eight additional DPP-4 inhibitors have received approval from different regulatory agencies worldwide and are widely prescribed for T2DM treatment, coinciding with ongoing efforts yielding at least ten novel DPP-4 inhibitors in clinical development [5]. Previously, we developed and discussed neogliptin, a novel DPP-4 inhibitor that demonstrated superior potency to sitagliptin, one of the most common DPP-4 inhibitors [13]. In this article, we utilised neogliptin as a template for further drug development, which yielded a novel compound **9a** with enhanced DPP-4 inhibiting activity, supported by structure–activity relationship (SAR) models and in vitro enzymatic assays.

## 2. Results

### 2.1. Chemical Synthesis

**Figure 2:**
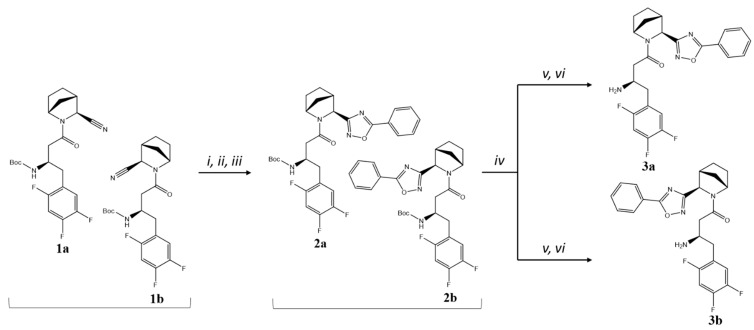
Synthesis of compounds **3a,b**. Reagents and conditions: (*i*) NH_2_OH×HCl, K_2_CO_3_, EtOH, overnight at *t* = 65–70 °C; (*ii*) PhCOOH, DIC, DCM, for 2 h at RT; (*iii*) Py, overnight at *t* = 110 °C; (*iv*) column chromatography on silica gel, Et_2_O/*n*–hexane 2:1; (*v*) TsOH, CH_3_CN, overnight at RT; (*vi*) column chromatography on silica gel, 1→10% EtOH in CHCl_3_.

5-phenyl-substituted 1,2,4-oxadiazoles **3a** and **3b** were synthesised from the corresponding nitriles **1a**,**b** (1:1 mixture of isomers) [13] by standard synthetic procedures from corresponding nitrile and acid components (Figure 2). Nitriles were converted into amidoximes and subsequently cyclised with benzoic acid to afford a mixture of intermediates **2a,b**, which were further separated by column chromatography, where diastereomer **2b** formed an upper spot on a TLC plate (as well as on the silica gel column) and was less soluble in Et_2_O or Et_2_O/*n*–hexane mixture than its counterpart **2a**. Moreover, **2b** was isolated as a crystalline powder, whereas **2a** was obtained as a solid foam. After Boc-deprotection and another column chromatography step, target compounds **3a** and **3b** were received as free amines.

**Figure 3:**
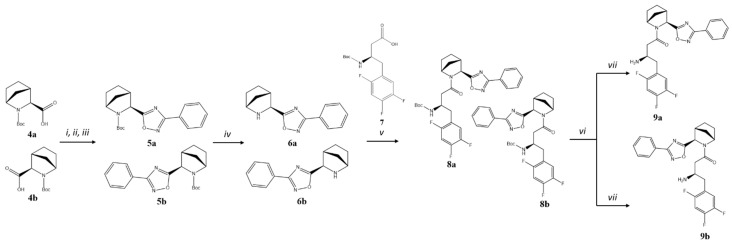
Synthesis of compounds **9a,b**. Reagents and conditions: (*i*) PhCN, NH_2_OH×HCl, K_2_CO_3_, EtOH, overnight at t = 65-70 °C; (*ii*) DIC, DCM, for 2 h at RT; *(iii*) Py, overnight at t = 110 °C; (*iv*) TsOH, CH_3_CN, overnight at RT; (*v*) BOP, DIPEA, DCM overnight at RT; (*vi*) column chromatography on silica gel, Et_2_O/*n*–hexane 2:1; Et_2_O/CHCl_3_ 0→2%; Et_2_O/DCM 2→5%; (*vii*) TsOH, CH_3_CN, overnight at RT, column chromatography on silica gel, 1 → 10% EtOH in CHCl_3_.

For the synthesis of 3-phenyl substituted 1,2,4-oxadiazoles **9a** and **9b,** another strategy was applied (Figure 3). Initially, starting from acids **4a,b** (1:1 mixture of isomers) [13], we prepared a mixture of isomeric amino-intermediates **6a,b** that were further acylated with Boc-protected (*R*)-3-amino-4-(2,4,5-trifluorophenyl)butanoic acid **7** using standard conditions. Diastereomer separation was performed by analogy with **2a,b**, and the separated intermediates **8a** and **8b** were subjected to Boc-deprotection, affording the corresponding target products **9a** and **9b.**

***tert*** **-Butyl {(2*R*)-4-[(3*S*)-3-(5-phenyl-1,2,4-oxadiazol-3-yl)-2-azabicyclo[2.2.1]heptan-2-yl]-4-oxo-1-(2,4,5-trifluorophenyl)butan-2-yl}carbamate (2a) and *tert*-Butyl {(2*R*)-4-[(3*R*)-3-(5-phenyl-1,2,4-oxadiazol-3-yl)-2-azabicyclo[2.2.1]heptan-2-yl]-4-oxo-1-(2,4,5-trifluorophenyl)butan-2-yl}carbamate (2b)**

To a solution of nitrile mixture **1a,b** (1 g, 2.3 mmol) in EtOH (30 mL), NH_2_OHxHCl (0.7 g, 10 mmol) and K_2_CO_3_ (0.83 g, 6 mmol) were added. The mixture was stirred overnight at 65–70 °C and then cooled to RT. After that, DCM (50 mL) was added, the precipitate was filtered, the solvent was evaporated, and the crude residue was used further.

To a solution of benzoic acid (0.31 g, 2.5 mmol) in DCM (20 mL), DIC (0.63 g, 5 mmol) was added. The mixture was stirred for 1 h at RT, and then the crude residue from the previous step was added, followed by another 1 h of stirring at RT. DCM was evaporated, and the residue was dissolved in pyridine (25 mL). The reaction mixture was stirred overnight at 110 °C, cooled to RT, and evaporated. The residue was dissolved in DCM (30 mL) and washed with 5% citric acid (3 × 10 mL) and 10% NaHCO_3_ (3 × 10 mL). The organic layer was dried over anhydrous Na_2_SO_4_, evaporated to 1–2 mL, and subjected to silica gel column chromatography purification (eluent: Et_2_O/*n*–hexane 2:1), leading to pure isomers **2a** (0.4 g) and **2b** (0.47 g). The total yield was 68%.

**2a** TLC (Et_2_O/*n*–hexane 3:1): Rf 0.4. ^1^H NMR (400 MHz, DMSO-*d6*), *δ* (ppm): 8.11, 8.06 (2 m, 2H, 2 rotameric forms, Ph), 7.76–7.56 (m overlapped, 3H, Ph), 7.94–7.36 (m, 1H, trifluoro-Ph), 7.32–7.22 (m, 1H, trifluoro-Ph), 6.70, 6.59, 6.35, 6.20 (4 m, 1H, NH-Boc, rotameric forms), 4.78, 4.65 (2 m, 1H, CHCN, 2 rotameric forms), 4.48, 4.42 (2 m, 1H, 2 rotameric forms), 4.08–3.97 (m, 1H), 2.93–2.54 (m overlapped, 4H), 2.33, 2.18–2.10 (2 m, 1H, 2 rotameric forms), 1.97–1.91, 1.85–1.57 (2 m, 5H), 1.47, 1.36 (2 m 1H, 2 rotameric forms), 1.26, 1.21 (2 s, 9H, Boc, 2 rotameric forms). ^13^C NMR (100 MHz, DMSO-*d6*), *δ* (ppm): 175.51, 174.83, 170.61, 170.51, 168.75, 167.98, 157.37–157.29, 154.95–154.85, 154.77, 154.70, 149.02–148.83, 146.76–146.58, 144.50–144.37, 133.51, 133.34, 129.60, 127.96, 127.80, 123.42, 123.35, 123.01–122.80, 119.67–119.20, 105.73–105.23, 77.65, 77.61, 58.95, 58.49, 57.89, 55.94, 47.40, 47.04, 43.34, 42.18, 38.40, 35.14, 33.34, 33.02, 32.74, 31.07, 29.52, 28.12, 28.06, 27.81, 26.80, 26.31. LC-MS: *m*/*z* 557 [M+H]^+^, 501 [M − t-Bu + H]^+^. Anal. calcd for C_29_H_31_F_3_N_4_O_4_: C, 62.58; H, 5.61; N, 10.07. Found: C, 62.76; H, 5.85; N, 9.84%.

The isomeric compound **2a** (0.2 g, 63%) was also prepared from pure isomer **1a** (0.25 g, 0.57 mmol) by the same procedure described above for **2a,b**. ^1^H NMR and LC-MS data, as well as Rf, are identical to those described above for **2a**.

**2b** TLC (Et_2_O/*n*–hexane 3:1): Rf 0.5. ^1^H NMR (400 MHz, DMSO-*d6*), *δ* (ppm): 8.12, 8.06 (2 m, 2H, 2 rotameric forms, Ph), 7.75–7.58 (m overlapped, 3H, Ph), 7.47–7.23, 7.22–7.12 (2 m, 2H, 2 rotameric forms, trifluoro-Ph), 6.80–6.69, 6.44–6.34 (2 m, 1H, NH-Boc, 2 rotameric forms), 4.97, 4.63 (2 m, 1H, CHCN, 2 rotameric forms), 4.48, 4.47 (2 m overlapped, 1H, 2 rotameric forms), 4.02 (m, 1H), 2.90–2.31 (m overlapped, 4H), 2.23–2.09 (m, 1H), 1.86–1.54 (m overlapped, 5H), 1.47, 1.35 (2 m, 1H, 2 rotameric forms), 1.26, 1.24 (2 s, 9H, Boc, 2 rotameric forms). ^13^C NMR (100 MHz, DMSO-*d6*), *δ* (ppm): 175.50, 174.80, 170.92, 170.47, 169.19, 168.23, 157.30–157.21, 154.82–154.76, 149.01–148.83, 146.89–146.57, 144.52–144.40, 133.50, 133.27, 129.64, 129.56, 127.92, 127.76, 123.45, 123.32, 123.06–122.75, 119.39–119.07, 105.59–105.22, 77.67, 77.50, 59.17, 58.56, 57.72, 55.89, 48.22, 47.47, 42.91, 42.15, 35.22, 33.32, 33.17, 32.87, 31.01, 29.72, 28.08, 28.05, 27.87–27.49, 26.82, 26.12. LC-MS: *m*/*z* 557 [M+H]^+^, 501 [M − t-Bu + H]^+^, 457 [M − Boc + H]^+^. Anal. calcd for C_29_H_31_F_3_N_4_O_4_: C, 62.58; H, 5.61; N, 10.07. Found: C, 62.77; H, 5.67; N, 9.76%.

**(3*R*)-3-Amino-1-[(3*S*)-3-(5-phenyl-1,2,4-oxadiazol-3-yl)-2-azabicyclo [2.2.1]heptan-2-yl]-4-(2,4,5-trifluorophenyl)butan-1-one (3a)**

To a solution of Boc-protected isomer **2a** (0.3 g, 0.54 mmol) in CH_3_CN (20 mL), p-TSAxH_2_O (0.21 g, 1.1 mmol) was added, and the resulting mixture was stirred overnight. After that, CH_3_CN was evaporated, and the residue was dissolved in CHCl_3_ and subjected to silica gel column chromatography purification (eluent: 0 → 10% EtOH in CHCl_3_), leading to **3a** in a free base form (0.22 g), yield 89%. ^1^H NMR (400 MHz, DMSO-*d6*), *δ* (ppm): 8.11, 8.05 (2 m, 2H, 2 rotameric forms, Ph), 7.74–7.58 (m, 3H, Ph), 7.48–7.38, 7.38–7.30 (2 m overlapped, 2H, 2 rotameric forms, trifluoro-Ph), 4.75, 4.65 (2 m, 1H, CHCN, 2 rotameric forms), 4.49, 4.46 (2 m, 1H, 2 rotameric forms), 3.4–3.2 (br, 2H, NH2), 3.29–3.22 (m, 1H), 2.75–2.69 (m, 1H), 2.64–2.52 (m overlapped, 2H, 2 rotameric forms), 2.37–2.21, 2.13–2.07 (2 m, 2H, 2 rotameric forms), 1.97–1.53 (m overlapped, 5H, 2 rotameric forms), 1.44, 1.35 (2 m, 1H, 2 rotameric forms). ^13^C NMR (100 MHz, DMSO-*d6*), *δ* (ppm): 175.45, 174.79 (NC=O), 170.72, 170.54, 169.99, 169.22 (two carbons of oxadiazole), 157.13–157.01, 154.72–154.60, 148.99–148.72, 147.06–146.91, 146.55–146.27, 144.66–144.51 (three quaternary CF-carbons of trifluorophenyl), 133.47, 133.26 (*para*-carbon of phenyl), 129.60, 129.56 (*meta*-carbon of phenyl), 127.91, 127.75 (*ortho*-carbon of phenyl), 123.84–123.55 (quaternary *ipso*-carbon of trifluorophenyl), 123.41, 123.29 (quaternary *ipso*-carbon of trifluorophenyl), 119.52–119.27 (*ortho*-carbon of trifluorophenyl), 105.72–105.22 (*meta*-carbon of trifluorophenyl), 79.23 (imp. of CHCl_3_), 59.03, 58.44, 57.69, 55.78 (two CH-carbons of azanorbornane), 49.85, 48.92, 48.30 (CHNH_2_-carbon), 43.25, 42.11, 41.74, 41.36, 35.36, 35.21, 35.11 (PhCH_2_ and COCH_2_ carbon of propane and CH-carbon of azanorbornane), 33.31, 31.10, 29.64, 28.58, 26.78, 26.28, 23.70 (three CH_2_-carbons of azanorbornane). LC-MS: *m*/*z* 457 [M+H]^+^. Anal. calcd for C_24_H_23_F_3_N_4_O_2_ ×0.6 CHCl_3_ × 0.3 C_6_H_13_NO (N-tert-butylacetamide): C, 56.36; H, 4.93; N, 10.70. Found: C, 56.51; H, 4.68; N, 10.46%.

**(3*R*)-3-Amino-1-[(3*R*)-3-(5-phenyl-1,2,4-oxadiazol-3-yl)-2-azabicyclo[2.2.1]heptan-2-yl]-4-(2,4,5-trifluorophenyl)butan-1-one (3b)**

**3b** (0.25 g, 88%) as free base was prepared from **2b** (0.35 g, 0.63 mmol) by analogy with 3a. ^1^H NMR (400 MHz, DMSO-*d6*), *δ* (ppm): 8.11–8.05 (m, 2H, Ph), 7.75–7.59 (m, 3H, Ph), 7.48–7.40, 7.30–7.19 (2 m, 2H, 2 rotameric forms, trifluoro-Ph), 4.89, 4.64 (2 m, 1H, CHCN, 2 rotameric forms), 4.50, 4.48 (2 m, 1H, 2 rotameric forms), 3.3–3.2 (br, 2H, NH2), 3.26–3.21, 3.19–3.13 (2 m overlapped, 1H, 2 rotameric forms), 2.75–2.66 (m, 1H), 2.65–2.56, 2.48–2.43 (2 m, 2H, 2 rotameric forms), 2.26–2.07, 1.98–1.84 (2 m, 2H, 2 rotameric forms), 1.82–1.51 (m overlapped, 5H), 1.45, 1.34 (2 m, 1H, 2 rotameric forms). ^13^C NMR (100 MHz, DMSO-*d6*), *δ* (ppm): 175.41, 174.79 (NC=O), 170.78, 170.52, 170.29, 169.26 (two carbons of oxadiazole), 157.10–157.00, 154.69–154.57, 148.84–148.70, 147.00–146.88, 146.53–146.26, 144.60–144.48 (three quaternary CF-carbons of trifluorophenyl), 133.42, 133.24 (*para*-carbon of phenyl), 129.54 (*meta*-carbon of phenyl), 127.83, 127.72 (*ortho*-carbon of phenyl), 123.82–123.42 (quaternary *ipso*-carbon of trifluorophenyl), 123.40, 123.21 (quaternary *ipso*-carbon of trifluorophenyl), 119.45–119.04 (*ortho*-carbon of trifluorophenyl), 105.68–104.79 (*meta*-carbon of trifluorophenyl), 79.20 (imp. of CHCl_3_), 59.00, 58.46, 57.80, 55.70 (two CH-carbons of azanorbornane), 49.22 (CHNH_2_-carbon), 43.19, 42.07, 41.79, 41.13, 35.78, 35.33, 35.06 (PhCH_2_ and COCH_2_ carbon of propane and CH-carbon of azanorbornane), 33.31, 31.02, 29.70, 26.74, 26.24 (three CH_2_-carbons of azanorbornane). LC-MS: *m*/*z* 457 [M+H]+. Anal. calcd for C_24_H_23_F_3_N_4_O_2_ ×0.5 CHCl_3_: C, 57.01; H, 4.59; N, 10.85. Found: C, 57.24; H, 4.38; N, 10.45%.

***tert-*** **Butyl (3*S*)-3-(5-phenyl-1,2,4-oxadiazol-3-yl)-2-azabicyclo[2.2.1]heptane-2-carboxylate (5a,b)**

The isomeric mixture **5a,b** (1.5 g, 66%) was prepared from **4a,b** (1.6 g, 6.6 mmol) and benzonitrile (1.0 g, 10 mmol) by analogy with **2a** and **2b**. DCM/Et_2_O 3:1 was used as eluent for column chromatography purification. ^1^H NMR (400 MHz, DMSO-*d6*), *δ* (ppm): 8.02–7.98 (m, 2H, Ph), 7.62–7.54 (m, 3H, Ph), 4.68, 4.67 (2 m, 1H, CHCN, 2 rotameric forms), 4.31, 4.22 (2 m, 1H, 2 rotameric forms), 2.80–2.73 (2 m overlapped, 1H, 2 rotameric forms), 2.05–1.96 (m, 1H), 1.81–1.56 (m overlapped, 4H), 1.43 (m, 1H), 1.41, 1.18 (2 s, 9H, Boc, 2 rotameric forms). ^13^C NMR (100 MHz, DMSO-*d6*), *δ* (ppm): 179.31, 179.08, 167.74, 167.72, 153.68, 152.08, 131.77, 131.75, 129.44, 129.39, 127.13, 127.08, 126.14, 126.13, 79.64, 79.25, 58.69, 58.48, 57.37, 55.95, 43.32, 42.85, 35.38, 34.71, 30.08, 29.87, 28.16, 27.81, 26.79, 26.68. LC-MS: *m*/*z* 286 [M − t-Bu + H]^+^, 242 [M − Boc + H]^+^. Anal. calcd for C_19_H_23_N_3_O_3_: C, 66.84; H, 6.79; N, 12.31. Found: C, 66.52; H, 6.75; N, 12.20%.

**(3*S*)-3-(3-phenyl-1,2,4-oxadiazol-5-yl)-2-azabicyclo[2.2.1]heptane** and **(3*R*)-3-(3-phenyl-1,2,4-oxadiazol-5-yl)-2-azabicyclo[2.2.1]heptane (6a,b)**

**6a,b** (0.8 g, 94%), obtained as a mixture of free base form isomers, was prepared from the Boc-protected mixture **5a,b** (1.2 g, 3.5 mmol) by analogy with the procedure described above for **3a**. ^1^H NMR (400 MHz, DMSO-*d6*), *δ* (ppm): 8.01–7.97 (m, 2H, Ph), 7.61–7.53 (m, 3H, Ph), 4.14 (m, 1H, CHCN), 3.44 (m, 1H), 2.77 (br, 1H, NH), 2.70 (m, 1H), 1.73–1.47 (m overlapped, 5H), 1.29 (m, 1H). ^13^C NMR (100 MHz, DMSO-d6), *δ* (ppm): 182.00, 167.38, 131.47, 129.26, 127.03, 126.47, 56.61, 55.32, 41.80, 35.44, 31.94, 27.85. LC-MS: *m*/*z* 242 [M+H]^+^. Anal. calcd for C_14_H_15_N_3_O: C, 69.69; H, 6.27; N, 17.41. Found: C, 69.90; H, 5.98; N, 17.20%.

***tert*** **-Butyl {(2*R*)-4-[(3*S*)-3-(3-phenyl-1,2,4-oxadiazol-5-yl)-2-azabicyclo[2.2.1]heptan-2-yl]-4-oxo-1-(2,4,5-trifluorophenyl)butan-2-yl}carbamate (8a) and *tert*-Butyl {(2*R*)-4-[(3*R*)-3-(3-phenyl-1,2,4-oxadiazol-5-yl)-2-azabicyclo[2.2.1]heptan-2-yl]-4-oxo-1-(2,4,5-trifluorophenyl)butan-2-yl}carbamate (8b)**

To the solution of acid **7** (0.73 g, 2.2 mmol) in DCM (30 mL), the following reagents were added: DIPEA (0.29 g, 2.2 mmol), BOP-reagent (0.98 g, 2.2 mmol), and amino-component **6a,b** (0.53 g, 2.2 mmol). The mixture was stirred overnight at RT and then washed with 5% citric acid (3 × 10 mL) and 10% NaHCO_3_ (3 × 10 mL). The organic layer was dried over anhydrous Na_2_SO_4_, evaporated to 1–2 mL, and subjected to silica gel column chromatography purification (eluent: Et2O/*n*–hexane 2:1), followed by two more column chromatography separations (eluents: 0 → 2% Et_2_O in CHCl_3_ and 2 → 5% Et_2_O in DCM), leading to pure isomers **8a** (0.43 g) and **8b** (0.5 g). The total yield was 76%.

**8a** TLC (Et_2_O/*n*–hexane 3:1): Rf 0.4. ^1^H NMR (400 MHz, DMSO-*d6*), *δ* (ppm): 8.00, 7.96 (2 m overlapped, 2H, 2 rotameric forms, Ph), 7.63–7.50 (m overlapped, 3H, Ph), 7.48–7.36 (m, 1H, trifluoro-Ph), 7.35–7.21 (m, 1H, trifluoro-Ph), 6.71, 6.61, 6.37 (3 m, 1H, NH-Boc, rotameric forms), 5.04, 4.80 (2 m, 1H, CHCN, 2 rotameric forms), 4.50, 4.49 (2 m overlapped, 1H, 2 rotameric forms), 4.07–3.96 (m, 1H), 2.94–2.81 (m, 1H), 2.76 (m, 1H), 2.68–2.53 (m, 2H), 2.14–2.07 (m, 1H), 1.85–1.37 (m overlapped, 6H), 1.25, 1.20, 1.18 (3 s, 9H, Boc, rotameric forms). ^13^C NMR (100 MHz, DMSO-*d6*), *δ* (ppm): 178.74, 178.34, 168.84, 168.10, 167.71, 167.57, 157.37–157.27, 154.94–154.76, 154.84, 149.03–148.84, 146.91–146.58, 144.51–144.39, 131.79, 131.67, 129.27, 127.18, 127.04, 126.14, 125.98, 122.90–122.70, 119.43–119.18, 105.73–105.23, 77.66, 58.69, 58.02, 57.85, 56.05, 47.36, 46.95, 43.78, 42.37, 38.32, 35.76, 33.88, 33.03, 32.84, 30.81, 28.10, 28.05, 27.79, 26.66, 26.09. LC-MS: *m*/*z* 557 [M+H]^+^, 501 [M − t-Bu + H]^+^, 457 [M − Boc + H]^+^. Anal. calcd for C_29_H_31_F_3_N_4_O_4_: C, 62.58; H, 5.61; N, 10.07. Found: C, 62.32; H, 5.56; N, 9.69%.

The isomeric compound **8a** (0.12 g, 49% over 3 steps) was prepared from pure isomeric acid **4a** (0.11 g, 4.6 mmol) by the same three-step procedure described above for **8a,b**. ^1^H NMR and LC-MS data, as well as Rf, are identical to those described above for **8a**.

**8b** TLC (Et_2_O/*n*–hexane 3:1): Rf 0.5. ^1^H NMR (400 MHz, DMSO-*d6*), *δ* (ppm): 8.02, 7.96 (2 m, 2H, 2 rotameric forms, Ph), 7.65–7.49 (m overlapped, 3H, Ph), 7.49–7.40, 7.39–7.34 (2 m overlapped, 1H, 2 rotameric forms, trifluoro-Ph), 7.34–7.22, 7.22–7.13 (2 m overlapped, 1H, 2 rotameric forms, trifluoro-Ph), 6.82–6.74, 6.46–6.40 (2 m, 1H, NH-Boc, 2 rotameric forms), 5.20, 4.77 (2 m, 1H, CHCN, 2 rotameric forms), 4.55, 4.52 (2 m overlapped, 1H, 2 rotameric forms), 4.07–3.95 (m, 1H), 2.95–2.67 (m overlapped, 3H), 2.64–2.55, 2.46–2.34 (2 m overlapped, 2H, 2 rotameric forms), 2.17–2.08 (m, 1H), 1.84–1.38 (m overlapped, 5H), 1.25, 1.23, 1.17 (3 s, 9H, Boc, rotameric forms). ^13^C NMR (100 MHz, DMSO-*d6*), *δ* (ppm): 178.66, 178.62, 169.28, 168.31, 167.80, 167.59, 157.36–157.18, 154.94–154.82, 154.86, 154.77, 149.21–148.88, 146.91–146.54, 144.40–144.37, 131.83, 131.64, 129.35, 129.26, 127.19, 127.04, 126.20, 125.96, 123.06–122.74, 119.41–119.12, 105.75–105.14, 77.69, 77.59, 58.95, 58.12, 57.74, 56.01, 48.21, 47.47, 43.49, 42.40, 35.86, 33.94, 33.17, 32.97, 30.76, 29.56, 28.09, 28.06, 27.75, 26.72, 25.99. LC-MS: *m*/*z* 557 [M+H]^+^, 501 [M − t-Bu + H]^+^, 457 [M − Boc + H]^+^. Anal. calcd for C_29_H_31_F_3_N_4_O_4_: C, 62.58; H, 5.61; N, 10.07. Found: C, 62.29; H, 5.28; N, 9.70%.

**(3*R*)-3-Amino-1-[(3*S*)-3-(3-phenyl-1,2,4-oxadiazol-5-yl)-2-azabicyclo[2.2.1]heptan-2-yl]-4-(2,4,5-trifluorophenyl)butan-1-one (9a)**

**9a** as free base (0.24 g, 89%) was prepared from **8a** (0.33 g, 0.6 mmol) by analogy with **3a**. ^1^H NMR (400 MHz, DMSO-*d6*), *δ* (ppm): 8.01, 7.96 (2 m, 2H, 2 rotameric forms, Ph), 7.63–7.50 (m, 3H, Ph), 7.47–7.31 (m overlapped, 2H, trifluoro-Ph), 5.04, 4.80 (2 m, 1H, CHCN, 2 rotameric forms), 4.52 (m, 1H), 3.4–3.2 (br, 2H, NH_2_), 3.29–3.22 (m, 1H), 2.87, 2.75 (2 m, 1H, 2 rotameric forms), 2.73–2.52 (m overlapped, 2H, 2 rotameric forms), 2.38–2.23 (m, 1H, 2 rotameric forms), 2.11–2.07 (m, 1H), 1.87–1.56 (m overlapped, 5H), 1.52–1.39 ppm (2 m, 1H, 2 rotameric forms). ^13^C NMR (100 MHz, DMSO-*d6*), *δ* (ppm): 178.77, 178.50 (NC=O), 170.05, 169.35, 167.73, 167.57 (two carbons of oxadiazole), 157.11–157.01, 154.72–154.60, 149.02–148.75, 147.07–146.91, 146.57–146.30, 144.68–144.52 (three quaternary CF-carbons of trifluorophenyl), 131.80, 131.64 (*para*-carbon of phenyl), 129.33, 129.28 (*meta*-carbon of phenyl), 127.16, 127.04 (*ortho*-carbon of phenyl), 126.15, 125.95 (quaternary *ipso*-carbon of phenyl), 123.76–123.47 (quaternary *ipso*-carbon of trifluorophenyl), 119.53–119.28 (*ortho*-carbon of trifluorophenyl), 105.76–105.26 (*meta*-carbon of trifluorophenyl), 79.24 (imp. of CHCl_3_), 58.75, 57.98, 57.68, 55.90 (two CH-carbons of azanorbornane), 49.86, 48.87, 48.20 (CHNH_2_-carbon), 43.75, 42.37, 41.54, 41.25, 35.77, 35.34, 35.16 (PhCH_2_ and COCH_2_ carbon of propane and CH-carbon of azanorbornane), 33.91, 30.84, 29.48, 28.59, 26.68, 26.08 (three CH2-carbons of azanorbornane). LC-MS: *m*/*z* 457 [M+H]+. Anal. calcd for C_24_H_23_F_3_N_4_O_2_ × 0.46 CHCl_3_ × 0.31 C_6_H_13_NO (N-tert-butylacetamide): C, 57.78; H, 5.06; N, 11.03. Found: C, 57.50; H, 5.30; N, 10.91%.

**(3*R*)-3-Amino-1-[(3*R*)-3-(3-phenyl-1,2,4-oxadiazol-5-yl)-2-azabicyclo[2.2.1]heptan-2-yl]-4-(2,4,5-trifluorophenyl)butan-1-one (9b)**

**9b** as free base (0.23 g, 88%) was prepared from **8b** (0.32 g, 0.58 mmol) by analogy with **3a**. ^1^H NMR (400 MHz, DMSO-*d6*), *δ* (ppm): 7.97 (m, 2H, Ph), 7.64–7.51 (m, 3H, Ph), 7.50–7.39, 7.32–7.15 (2 m, 2H, 2 rotameric forms, trifluoro-Ph), 5.17, 4.78 (2 m, 1H, CHCN, 2 rotameric forms), 4.55, 4.52 (2 m, 1H, 2 rotameric forms), 3.40–3.20 (br, 2H, NH2), 3.27–3.20 (m, 1H), 2.88, 2.77–2.66 (2 m, 2H, 2 rotameric forms), 2.63–2.53 (m, 1H), 2.32–2.23, 2.22–2.15 (2 m, 1H, 2 rotameric forms), 2.13–2.05 (m, 1H), 1.94–1.59 (m overlapped, 5H), 1.51, 1.40 (2 m, 1H, 2 rotameric forms). ^13^C NMR (100 MHz, DMSO-d6), *δ* (ppm): 178.78, 178.60 (NC=O), 170.37, 169.37, 167.68, 167.56 (two carbons of oxadiazole), 157.13–157.04, 154.72–154.61, 148.89–148.75, 147.06–146.94, 146.43–146.29, 144.66–144.54 (three quaternary CF-carbons of trifluorophenyl), 131.79, 131.67 (*para*-carbon of phenyl), 129.30, 127.10 (*meta*-carbon of phenyl), 127.02 (*ortho*-carbon of phenyl), 126.14, 125.87 (quaternary *ipso*-carbon of phenyl), 123.74–123.46 (quaternary *ipso*-carbon of trifluorophenyl), 119.51–119.04 (*ortho*-carbon of trifluorophenyl), 105.78–105.28 (*meta*-carbon of trifluorophenyl), 79.24 (imp. of CHCl_3_), 58.81, 58.00, 57.75, 55.84 (two CH-carbons of azanorbornane), 49.29, 49.03 (CHNH_2_-carbon), 43.63, 42.33, 41.55, 40.98, 35.75, 35.33 (PhCH_2_ and COCH_2_ carbon of propane and CH-carbon of azanorbornane), 33.94, 30.76, 29.57, 28.60, 26.66, 26.05 (three CH2-carbons of azanorbornane). LC-MS: *m*/*z* 457 [M+H]^+^. Anal. calcd for C_24_H_23_F_3_N_4_O × 0.16 CHCl_3_ × 0.21 C_6_H_13_NO (N-tert-butylacetamide): C, 61.09; H, 5.22; N, 11.80. Found: C, 61.30; H, 5.27; N, 11.60%.

### 2.2. Molecular Modelling

**Figure 4:**
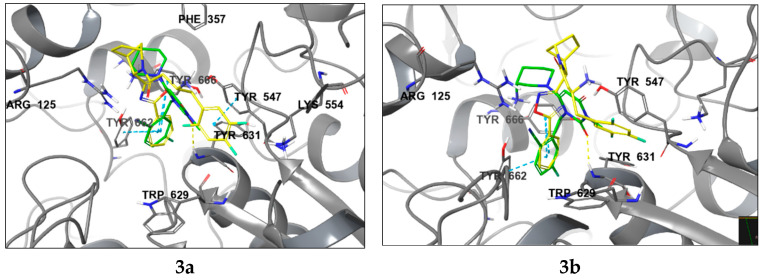

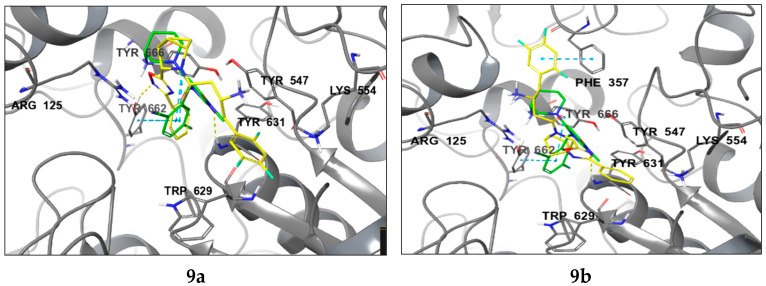
The binding poses of compounds **3a**, **3b**, **9a**, and **9b** (shown in yellow) in contrast to trelagliptin (green) when bound to DPP-4. Dashed lines represent various interactions: pi-stacking (blue), hydrogen bonds (yellow), and strained contacts (orange). Detailed high-quality figures are available in the Supplementary Information (SI).

The molecular docking analysis of compounds **3a**, **3b**, **9a**, and **9b** revealed that **9a** exhibited the highest potency against DPP-4, as indicated by the docking score (GScore) in Table 1. This observation was further supported by the consistent alignment of pharmacophore characteristics with those of the reference compound, trelagliptin (Figure 4). Compound **9a** exhibits a more densely populated network of lipophilic contacts, as evidenced by lower values of the corresponding increment in the scoring function. Upon complex formation, the trifluorophenyl group in **9a** establishes tighter interactions with Trp627/629, and contact with Tyr752 becomes apparent, accounting for the increased lipophilic contact. Notably, the oxadiazole scaffold in **9a** undergoes a 90-degree rotation relative to its analogous counterpart in **3a**, facilitating the formation of a new hydrogen bond with Tyr662. It is also noteworthy that compounds **3b** and **9b** exhibited a distinct binding pose compared to **9a** and trelagliptin, along with inferior GScore values and strain energy parameters in the ligand–protein complex (ΔG strain). It was revealed that **9a** induces minimal structural rearrangements in the DPP-4 protein compared to its isomers.

**Figure 5:**
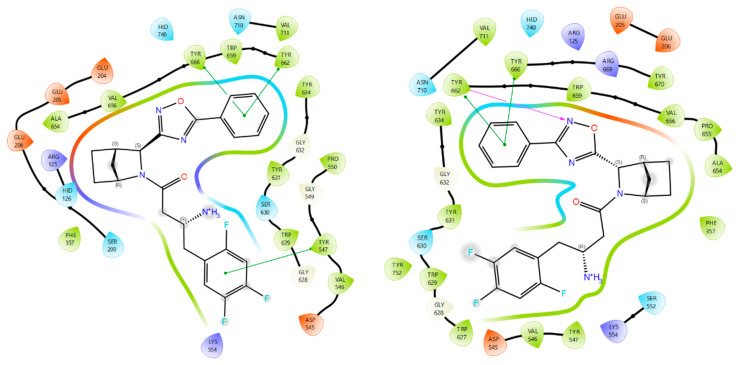

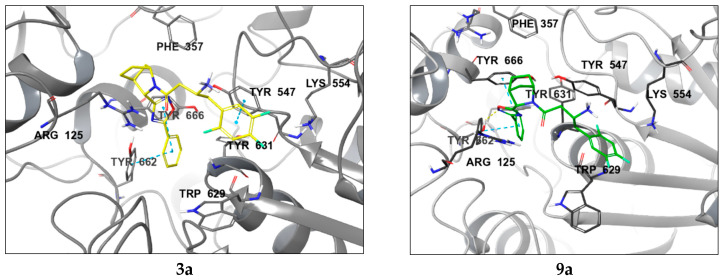
Ligand interaction diagrams and the binding poses of compounds **3a** and **9a** in the active cavity of DPP-4. Dashed lines represent various interactions: pi-stacking (blue), hydrogen bonds (yellow), and strained contacts (orange). Detailed high-quality figures are available in the Supplementary Information (SI).

Compounds **3a** and **9a** showcase an increased prevalence of aromatic substituents, thereby intensifying the involvement of stacking interactions within the molecular system. As shown in Figure 5, these interactions simultaneously involve three tyrosine residues: Tyr547, Tyr662, and Tyr666. A notable repositioning of the trifluorophenyl fragment is observed, facilitating interactions with Trp629 and Tyr547 upon incorporating a phenyl substituent into the oxadiazole heterocycle. This spatial rearrangement results in the mediation of additional lipophilic contacts, consequently enhancing the site specificity and affinity of compounds **3a** and **9a** compared to neogliptin (refer to the Supplementary Materials for a detailed comparison of binding poses). Furthermore, interactions with charged amino acid residues, specifically Arg125 and Arg669, oriented towards the electron density of the heterocycle, are maintained. This preservation ensures optimal molecular recognition of lipophilic cavities, thereby contributing to the overall binding effectiveness of the compounds.

### 2.3. X-Ray Diffraction Study

**Figure 6:**
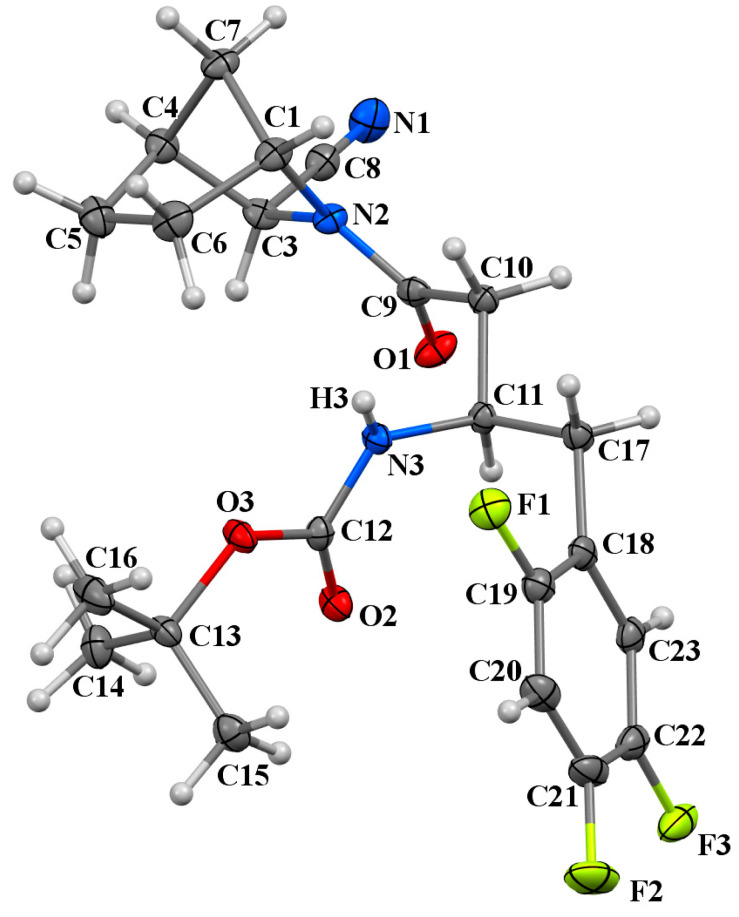
The molecular structure of **1a** showing the labelling scheme: non-hydrogen atoms (C1-23 are carbons shown in grey, N1-3 are nitrogen atoms shown in blue, O1-3 are oxygen atoms shown in red, F1-3 are fluorine atoms shown in lime) are represented as 30% probability displacement ellipsoids. Hydrogen atoms are depicted as small white spheres with arbitrary radii.

The stereoselective synthesis of the respective starting materials, **1a** and **4a**, which correspond to the target compounds, **3a** and **9a**, was conducted to establish precise structural assignments for the isolated isomeric compounds. These starting compounds were synthesised following the methodology outlined in [13]. In the case of **1a,** single crystals suitable for X-ray diffraction analysis were successfully grown. The crystallographic analysis validated the structure of the intended isomer (Figure 6).

### 2.4. Inhibitory Activity Assays

The assessment of DPP-4 enzyme inhibitory activities for the synthesised compounds was performed using protocols similar to those previously described in the literature [13,14]. For the determination of IC_50_ values, inhibitory assays were conducted employing recombinant DPP-4 enzyme D4943, the chromogenic substrate Gly-Pro-*p*NA, and a buffer system (50 mM Tris-HCl, 50 mM NaCl, 0.01% Triton, pH = 7.6). Following a brief incubation period (37 °C for 30 min), absorbance at 405 nm was measured. Initial optimisation of the procedure utilised reference compounds. Each inhibitor was analysed in a dilution range spanning from 10^−4^ to 10^−11^ M. Certified samples of commercially available DPP-4 inhibitors (vildagliptin, sitagliptin, alogliptin, and linagliptin) with known IC_50_ values, as well as precursor nitriles (including neogliptin), were utilised as reference compounds [13]. Correspondingly, the analysis of inhibition activity for related enzymes was executed using DPP-8 and DPP-9 inhibition assays, where the compounds were studied in the dilution range 10^−2^ to 10^−8^ M (Table 2).

## 3. Discussion

Oxadiazoles are heterocyclic compounds that have gathered substantial attention in medicinal chemistry due to their diverse pharmacological activities and structural versatility. Various agents bearing oxadiazole moieties have been explored as potential drug candidates in different therapeutic areas, including antimicrobial, anti-inflammatory, and anticancer agents [15,16,17,18]. The considerable potential of oxadiazole-containing scaffolds for DPP-4 inhibition was previously demonstrated by Nordhoff et al. [14,19], where such oxadiazole-containing DPP-4 inhibitors exhibited improved potency and metabolic stability. Following this approach, we modified the nitrile group of neogliptin, resulting in four isomeric oxadiazole derivatives. Similarly to the findings of Nordhoff et al. [14], some of the synthesised oxadiazoles were shown to be potent inhibitors of DPP-4, outperforming their precursor nitriles. Some differences among the synthesised diastereomers were observed, with RS-stereoisomers (**3a** and **9a**) being more potent over RR-isomers (**3b** and **9b**). Interestingly, closely related oxadiazole-substituted pyrrolidines, studied as novel anthelmintics, demonstrated that only the *S*-enantiomer of 3-phenyl-5-(pyrrolidine-2-yl)-1,2,4-oxadiazole derivative exhibited the inhibitory activity on nematode motility and development, whereas the R-enantiomer remained inactive [20]. Our study revealed that only **9a** exhibited a higher DPP-4 inhibitory activity than neogliptin, suggesting the importance of the 3-phenyl substitution in the 1,2,4-oxadiazole heteroatom orientation.

While DPP-4 inhibitors are generally well tolerated and effective in managing T2DM, inhibiting DPP-8 and DPP-9 has raised concerns. DPP-8 and DPP-9 have broader substrate specificities compared to DPP-4 and are involved in various biological processes, including immune response regulation. The previous literature suggests that inhibiting DPP-8 and DPP-9 may be associated with some adverse effects, such as haematological and gastrointestinal issues [9,21]. In our study, all of the tested oxadiazole-derivatives showed feeble DPP-8 and DPP-9 inhibitory activity in the concentration range 10^−3^–10^−6^ M. This is below the estimated therapeutic range and is consistent with the data in the literature for already known DPP-4 inhibitor compounds, making it possible to predict the absence of side effects associated with the undesirable inhibition of homologous enzymes DPP-8 and DPP-9.

It is also crucial to highlight that we used distinct diastereomers to measure the DPP-4 inhibitory activity of the synthesised compounds. Acid **4a** is a precursor in the synthetic route leading to **1a** [13]; therefore, we can reasonably infer the same isomeric structure for **4a**, as confirmed by the X-ray diffraction study for **1a**. These distinct diastereomers served as precursors for the syntheses of **3a** and **9a**, employing methodologies analogous to those delineated in Figure 1 and Figure 2. A compelling indication of the exclusive production of the targeted diastereomers was validated through the absence of corresponding spots on the TLC plate for **2b** and **8b**, which were perfectly visible in diastereomeric mixtures These observations and NMR results further support the successful synthesis of the intended isomers **3a** and **9a**.

## 4. Materials and Methods

### 4.1. Chemical Synthesis

All starting reagents were obtained from reliable commercial vendors, mostly Sigma-Aldrich (St. Louis, United States), Merck (Darmstadt, Germany), and Acros (Geel, Belgium), and used without further purification. Intermediates and final compounds were isolated using column chromatography on silica gel. Compounds were only used for biological evaluation if the purity was ≥95%.

### 4.2. Molecular Modelling

The docking procedure was performed using the Schrödinger Glide module in standard precision mode [22]. The docking grid was calculated according to native ligand dimensions using the DPP-4 PDB model (5KBY) [23]. The docking area was limited per reference ligand size, with 7 Å as a buffer zone. Grid spacing was set at 0.375 Å, VdW radii cut-off 0.8 Å. Several optional constraints were added: nitrile group orientation (reference—trelagliptin), hydrophobic attraction—aromatic and aliphatic moiety (trelagliptin). The generation of docking solutions was performed using the Glide module of Schrödinger Suite (version 2022-4) in standard precision mode with 0.8 Å VdW radius and with the previously mentioned optional constraints. The docking protocol was validated by redocking reference compounds (sitagliptin, trelagliptin, vildagliptin). For each inhibitor, 45 docking solutions were generated, and the best 15 were used for binding mode analysis using GlideScore and EModel values to control target affinity. Optimal binding poses were selected per cluster with an RMSD of less than 1.5 Å. The binding poses and calculated parameters of the reference ligands were taken as a control. Free Gibbs energy (∆G) was calculated using the MM-GBSA method [24], implemented in Schrödinger Suite v.2022-4, module Prime. All results were processed using the Maestro molecular modelling interface (Schrödinger Suite v.2022-4). All protein–ligand complexes were prepared and refined using Schrödinger Protein Prepwizard [25]. This procedure was essential to fix missing amino acid sidechains, incorrect bond orders, and correct protonation states. Optimal binding poses were selected by cluster RMSD less than 1.5 Å. The binding parameters of the reference ligand were chosen as a control.

### 4.3. Structure and Purity Confirmation

LC/MS analysis was performed on an 1100 LC (Agilent Technologies Inc., Santa Clara, CA, USA) with ELSD, UV (DAD 200–400 nm) and mass detection (1100 LCMSD, Agilent Technologies, APCI and ES positive ionisation). The most used column was the Onix C18 50 × 4.6 mm; eluent 1—0.1% TFA in water; eluent 2—0.1% TFA in acetonitrile, gradient—eluent 1—2.9 min, eluent 2—0.2 min, eluent 1—rinsing, flow rate 3.75 mL/min. 1H, 13C, COSY and HSQC NMR spectra were registered on the spectrometer Bruker DRX 400 (400.13 MHz for protons, 100.61 MHz for carbons). DMSO-d6 was used as a solvent. Elemental analysis was performed on the Vario MICRO cube CHNS analyser (Elementar Analysensysteme GmbH, Hanau, Germany).

### 4.4. X-Ray Diffraction Study

Crystals of **1a** (C_22_H_26_F_3_N_3_O_3_, M = 437.46) are orthorhombic, space group *P*2_1_2_1_2_1_, at 120K *a* = 5.5917(13), *b* = 10.260(2), *c* = 38.079(8) Å, V = 2184.7(9) Å^3^, Z = 4, *d*_calc._ = 1.330 g/cm^3^, *μ* = 1.06 cm^−1^. Data collection was carried out with a Bruker SMART APEX II diffractometer, *λ*(MoKα) = 0.71073 Å, ω-scan technique, T = 120(2) K, 5472 independent reflections (*R*_int_ = 0.0663) with *θ_max_* = 28.346° collected and used in refinement. The structure was solved by direct methods and refined by the full matrix least-squares technique against *F*^2^ with the anisotropic thermal parameters for all non-hydrogen atoms. The hydrogen atom of the N-H group was located from the Fourier maps and isotropically refined without restrictions; the remaining hydrogen atoms were placed geometrically and included in the structure factor calculations in a riding motion approximation with isotropic thermal parameters *U*_iso_(H) = 1.5*U*_eq_(C) for the hydrogen atoms of methyl groups and *U*_iso_(H) = 1.2*U*_eq_(C) for other carbon atoms. The refinement converged to *wR_2_* = 0.0912 and GOF = 1.010 for all independent reflections (*R_1_* = 0.0448 was calculated against *F* for 4015 observed reflections with *I* > 2σ(*I*)). All calculations were performed using the SHELXL programme package [26].

### 4.5. Inhibitory Activity Evaluation

For the in vitro assays, we used the substrate Gly-Pro-*p*-nitroanilide (H-Gly-Pro *p*NA HCl, G0513) and recombinant dipeptidyl peptidase-4 enzyme (D4943), which were purchased from Sigma-Aldrich (St. Louis, MO, USA). Samples containing DPP-4 (0.0015 U/well) and varying concentrations of test compounds were incubated with the chromogenic peptide substrate, G0513 (90 µg/well), in a total volume of 100 µL of buffer system (50 mM Tris-HCl, 50 mM NaCl, 0.01% Triton, pH = 7.6). The mixtures were incubated at 37 ◦C for 30 min, and the absorbance at 405 nm was measured using the microplate reader ChemWell (Awareness Technology Inc., Palm City, FL, USA). The DPP-4 inhibitory activities for each test compound were calculated. IC_50_ values were obtained using GraphPad Prism 8 software. The inhibition activity analysis of the related enzymes was performed using the “Fluorogenic DPP-8 Assay Kit” and “Fluorogenic DPP-9 Assay Kit”, both purchased from BPS Bioscience (San Diego, CA, USA). The dilution range for every compound was 10^−2^–10^−8^ M.

## 5. Conclusions

In this article, we show that pseudo peptides containing (*R*)-3-amino-4-(2,4,5-trifluorophenyl)butanoic acid and bicyclic amino moiety (2-aza-bicyclo[2.2.1]heptane) with 1,2,4-oxadiazole substituents are potent DPP-4 inhibitors. The strategic modification of neogliptin and the emphasis on stereochemistry have yielded compound **9a** with enhanced inhibitory activity against DPP-4 while minimising the risk of inhibiting DPP-8 and DPP-9. The comprehensive evaluation, combining computational and experimental approaches, provides valuable information for developing DPP-4 inhibitors with improved efficacy and safety profiles. Our study underscores the promising role of **9a** with potential applications in managing T2DM as it emerged as the most promising candidate for further optimisation and subsequent animal studies.

## 6. Patents

Patent (Russian Federation), Issue No. 2018134266, date of issue 28 September 2018, date of registration in the State Register of Inventions (RU) 24 January 2020, date of expiry 28 September 2038. Dipeptidyl peptidase-4 inhibitor for treating type 2 diabetes mellitus, compounds (versions)//Patentee: Neobiotek LLC (Moscow, Russia). Authors: Trukhan V.M., Zinevich T.V., Maslov I.O., Kirichenko O.G.

## Figures and Tables

**Table 1:** The scoring outcomes of **3a**, **3b**, **9a**, and **9b** in comparison with trelagliptin. The lead compound is highlighted in green.

Structure	GScore (kcal/mol)	LE (kcal/atom)	Lipo (kcal/mol)	ΔG Lipo	ΔG	ΔG Strain
Trelagliptin	−8.33	−0.32	−1.27	−2.20	−30.19	0.01
**3a**	−6.54	−0.20	−2.04	−8.16	−29.17	−4.96
**3b**	−5.85	−0.18	−1.35	−2.68	−32.96	−1.19
**9a**	−6.37	−0.19	−2.78	−8.04	−31.11	−4.26
**9b**	−6.01	−0.18	−1.33	−2.96	−29.72	2.32

**Table 2:** DPP-4-, DPP-8-, and DPP-9-inhibitory activities of target compounds **3a**, **3b**, **9a**, and **9b** in comparison with neogliptin.

Structure	DPP-4 IC_50_, nM	DPP-8 IC_50_, nM	DPP-9 IC_50_, nM
Neogliptin [13]	16.8	>1000	>1000
**3a**	21.6	>1000	>1000
**3b**	>1000	>1000	>1000
**9a**	4.3	>1000	>1000
**9b**	>1000	>1000	>1000

## Data Availability

The data presented in this study are available in the article and the Supplementary Materials. CCDC 2307518 contains the supplementary crystallographic data for this paper. These data can be obtained free of charge via www.ccdc.cam.ac.uk/data_request/cif, accessed on 20 March 2025, or by emailing data_request@ccdc.cam.ac.uk, or by contacting The Cambridge Crystallographic Data Centre, 12 Union Road, Cambridge CB2 1EZ, UK; Fax: +44 1223 336033.

## Supplementary Materials

The following supporting information can be downloaded at: https://www.mdpi.com/article/10.3390/ph18050642/s1.
