# Supplementary material for: 3-(3-Azabicyclo[2, 2, 1]heptan-2-yl)-1,2,4-oxadiazoles as Novel Potent DPP-4 Inhibitors to Treat T2DM
Source: Pharmaceuticals (Basel). 2025 Apr 28;18(5):642. doi: 10.3390/ph18050642 (PMC12114571; doi:10.3390/ph18050642)
Supplement: Supplementary file 1 [file pharmaceuticals-18-00642-s001.zip › LCMS/3b_LCMS.pdf]

===

Injection Date : 22/6/23 16:41:00 PM Seq. Line : 43
Sample Name : ULZ-553 Location : P1-B-11
Acq. Operator : #6 Inj : 1
Acq. Instrument : Instrument 1 Inj Volume : Inj prog
Method : C:\HPCHEM\1\METHODS\1PH08.M
Last changed : 19/6/23 12:42:13 PM by #6
Column: Onyx C18 50x2.1mm | 0.80ml/min | Columns Reg Valve
Gradient: "A"->@2.0min->"B"(Hold 0.6min)->@0.05min->"A"(Hold 0.95min)->PostRun

===

Instrument Conditions	At Start	At Stop	
Pressure	122.7	69.3	bar
Flow	0.800	0.800	ml/min

Detector Lamp Burn Times:	Current On-Time	Accumulated On-Time	
DAD 1, UV Lamp	3.37	87816.0	h
DAD 1, Visible Lamp	OFF	13251.8	h

Solvent Description :
PMP1, Solvent A : 0.1%TFA in Acn/H2O (2.5:97.5)
PMP1, Solvent B : 0.1%TFA in AcN
PMP1, Solvent C : 0.1%FA in Acn/H2O (2.5:97.5)
PMP1, Solvent D : 0.1%FA in AcN

===

MSD parameters
Tune file name : C:\HPCHEM\1\1956ATUN\atunes.tun
Ionization mode : APCI

MSD Instrument Conditions	At Start	At Stop	
Quad Temp	99	99	C
Gas Temp	350	350	C
Vaporizer	332	321	C
RoughVac	3	3	Torr
HighVac	9.8E-006	9.8E-006	Torr
CapCur	78	980	nA
ChamCur	4	0	µA
CoronaVol	2902	39	Volt
DryingGas	4	4	l/min
Neb Pres	50	50	psig
Turbo1Spd	99	99	%
Turbo1Pwr	106	106	W
RF Drive	0.0E-001	0.0E-001	%
Qd TpDrv	8	10	%
Gas TpDrv	16	16	%
Vap TpDrv	40	46	%
Neb PrDrv	41	41	%
Gas FlDrv	53	53	%
DelaySens	0.0E-001	0.0E-001	V
Aux Input	0.0E-001	0.0E-001	V
Other Det	0.0E-001	0.0E-001	V

===

MSD tuning (calibration) parameters

Ionization polarity : Positive
Skim1 : Not Applicable
Skim2 : 8.0 V
Ion Energy : 5.0 V
Lens1 : 3.1 V
Lens2 : 36 V
Iris : -200 V
HED : 10000 V
Width Gain : -893
Width Offset : Variable

Mass	Value
121.05	-154
622.03	-154
922.01	-154

Mass Gain : -36.15
Mass Offset : Variable

Mass	Value

```
                                 121.05       :   0.624
                                 622.03       :   0.672
                                 922.01       :   0.624
                                 --------------------------
Quad DC                       :  0.00 V
Octopole Peak                 :  650 V
Octopole Knee                 :  Not Applicable
Lens2DC                       :  Not Applicable
L2RFEn                        :  Not Applicable
L2RFPh                        :  Not Applicable
L2RFAmp                       :  Not Applicable
Mass Filter                   :  Gaussian
Mass Filter Width             :  0.30 Da
Time Filter                   :  Gaussian
Time Filter Width             :  0.030 minutes
```

===

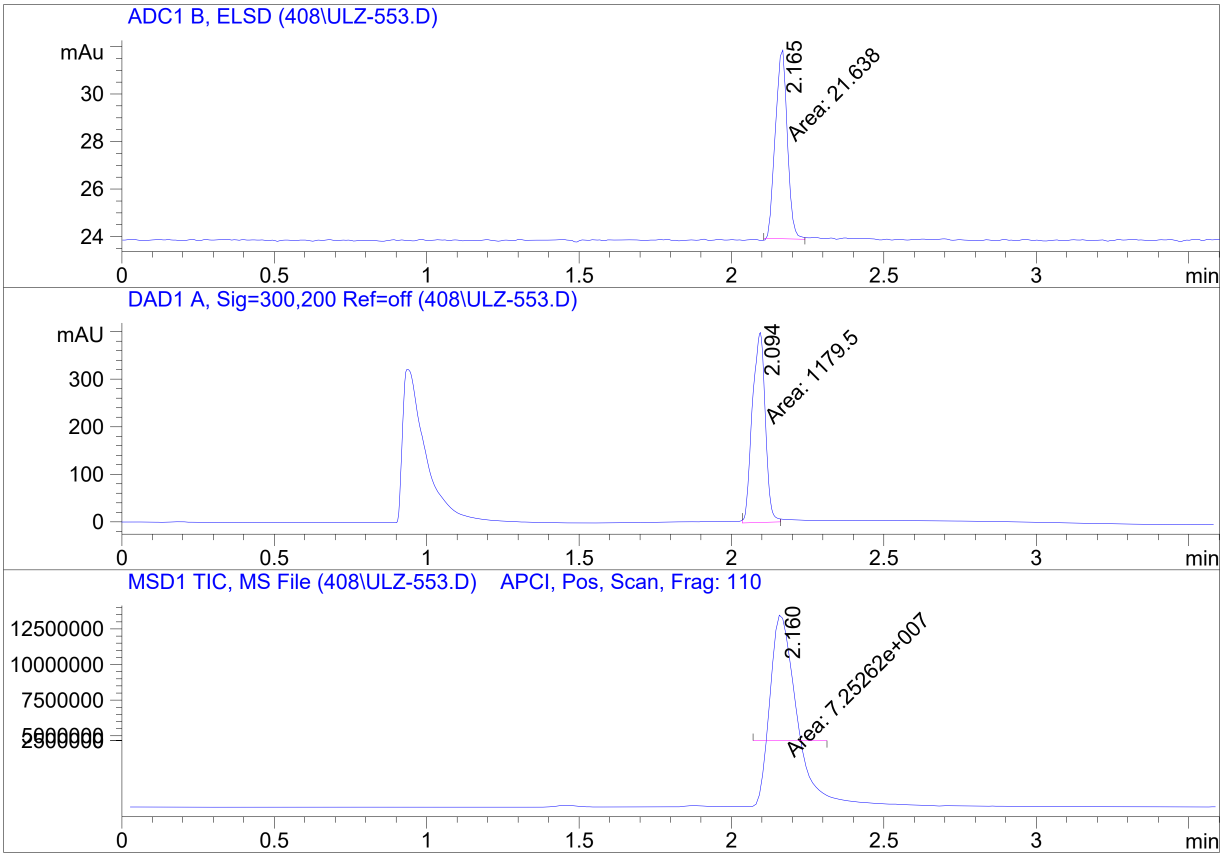

===

Area Percent Report

===

Sorted By : Signal
Multiplier : 1.0000
Dilution : 1.0000
Use Multiplier & Dilution Factor with ISTDs

Signal 1: ADC1 B, ELSD

Peak #	RetTime [min]	Type	Width [min]	Area [mAu*s]	Height [mAu]	Area %
1	2.165	MM	0.0442	21.63797	8.16249	100.0000

Totals : 21.63797 8.16249

Signal 2: DAD1 A, Sig=300,200 Ref=off

Peak #	RetTime [min]	Type	Width [min]	Area [mAU*s]	Height [mAU]	Area %
1	2.094	MM	0.0490	1179.50073	400.82391	100.0000

Totals : 1179.50073 400.82391

Signal 3: MSD1 TIC, MS File

Peak #	RetTime [min]	Type	Width [min]	Area	Height	Area %
1	2.160	MM	0.0921	7.25262e7	1.31286e7	100.0000

Totals : 7.25262e7 1.31286e7

===

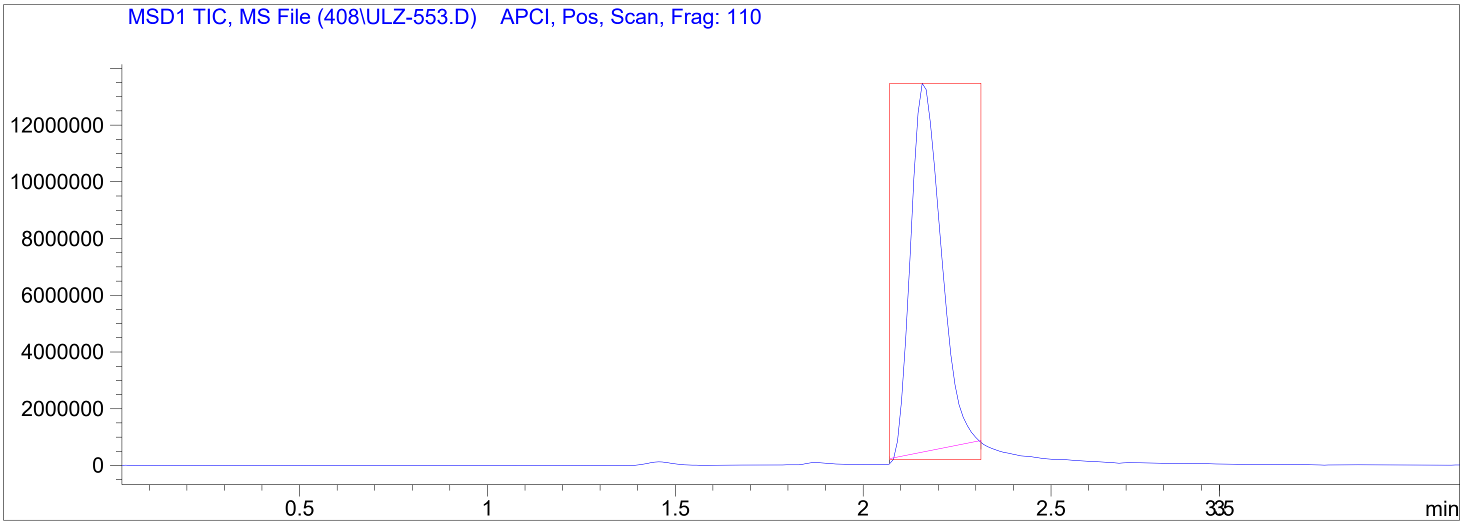

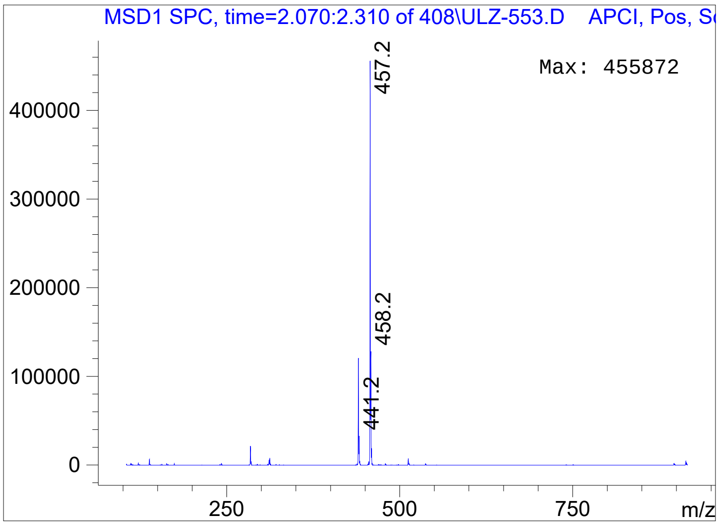

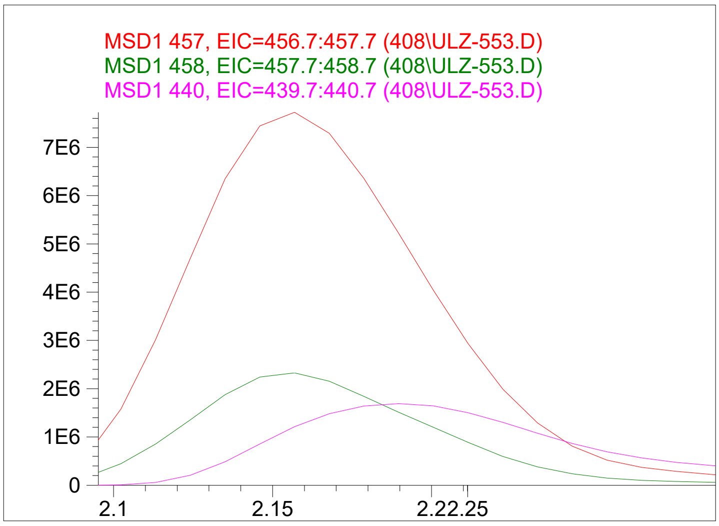


```
Peak #1 at  2.160 min ( 2.071 to  2.314 min)
-> The analysis found 2 components, indicating an impure peak. <-

Component 1:  Peak at Scan 195.9. Top ions are  457 458
Component 2:  Peak at Scan 199.0. Top ions are  440
```

*** End of Report ***
