# Supplementary material for: 3-(3-Azabicyclo[2, 2, 1]heptan-2-yl)-1,2,4-oxadiazoles as Novel Potent DPP-4 Inhibitors to Treat T2DM
Source: Pharmaceuticals (Basel). 2025 Apr 28;18(5):642. doi: 10.3390/ph18050642 (PMC12114571; doi:10.3390/ph18050642)
Supplement: Supplementary file 1 [file pharmaceuticals-18-00642-s001.zip › LCMS/5a,b_LCMS.pdf]

```
=====================================================================
Injection Date  : 16/11/2022 15:01:25          Seq. Line :  21
Sample Name     : ULZ-518                       Location : P1-E-12
Acq. Operator   : 1                                  Inj :   1
Acq. Instrument : Instrument 1                Inj Volume : Inj prog
Method          : C:\HPCHEM\1\METHODS\1PH08.M
Last changed    : 20/10/2022 10:26:39  by 1
Column: Onyx C18 50x2.1mm | 0.80ml/min | Columns Reg Valve
Gradient: "A"->@2.0min->"B"(Hold 0.6min)->@0.05min->"A"(Hold 0.95min)->PostRun
=====================================================================

Instrument Conditions   :    At Start          At Stop
Pressure                :      92.4             46.7   bar
Flow                    :       0.800            0.800 ml/min

Detector Lamp Burn Times: Current On-Time  Accumulated On-Time
DAD 1, UV Lamp          :      29.81           53439.5   h
DAD 1, Visible Lamp     :      OFF             3915.7   h

Solvent Description     :
PMP1, Solvent A         : 0.1%TFA in Acn/H2O (2.5:97.5)
PMP1, Solvent B         : 0.1%TFA in AcN
PMP1, Solvent C         : 0.1%FA in Acn/H2O (2.5:97.5)
PMP1, Solvent D         : 0.1%FA in AcN
=====================================================================
MSD parameters
Tune file name              :    C:\HPCHEM\1\1956ATUN\atunes.tun
Ionization mode             :    API-ES

MSD Instrument Conditions   :      At Start        At Stop
Quad Temp                   :            99              99 C
Gas Temp                    :           350             350 C
RoughVac                    :             2               2 Torr
HighVac                     :      1.2E-005        1.2E-005 Torr
CapCur                      :            55             525 nA
ChamCur                     :      8.6E-001        2.4E-001 µA
DryingGas                   :             8               8 l/min
Neb Pres                    :            40              40 psig
Turbo1Spd                   :            99              99 %
Turbo1Pwr                   :            98              97 W
RF Drive                    :      0.0E-001        0.0E-001 %
Qd TpDrv                    :             8               8 %
Gas TpDrv                   :            25              25 %
Neb PrDrv                   :            45              45 %
Gas FlDrv                   :            61              58 %
DelaySens                   :      -9.9E-002       -9.9E-002 V
Aux Input                   :      0.0E-001        0.0E-001 V
Other Det                   :      0.0E-001        0.0E-001 V
=====================================================================
MSD tuning (calibration) parameters

Ionization polarity         :    Positive
Skim1                       :    Not Applicable
Skim2                       :    8.0 V
Ion Energy                  :    5.0 V
Lens1                       :    3.6 V
Lens2                       :    20 V
Iris                        :    20 V
HED                         :    10000 V
Width Gain                  :    -294
Width Offset                :    Variable
                                 Mass         :   Value
                                 -------------------------
                                 118.08       :   -108
                                 622.03       :   -108
                                 922.01       :   -108
                                 -------------------------
Mass Gain                   :    -39.20
Mass Offset                 :    0.642
Quad DC                     :    0.00 V
Octopole Peak               :    650 V
Octopole Knee               :    Not Applicable
Lens2DC                     :    Not Applicable
L2RFEn                      :    Not Applicable
L2RFPh                      :    Not Applicable
```

L2RFAmp	:	Not Applicable
Mass Filter	:	Gaussian
Mass Filter Width	:	0.30 Da
Time Filter	:	Gaussian
Time Filter Width	:	0.030 minutes

===

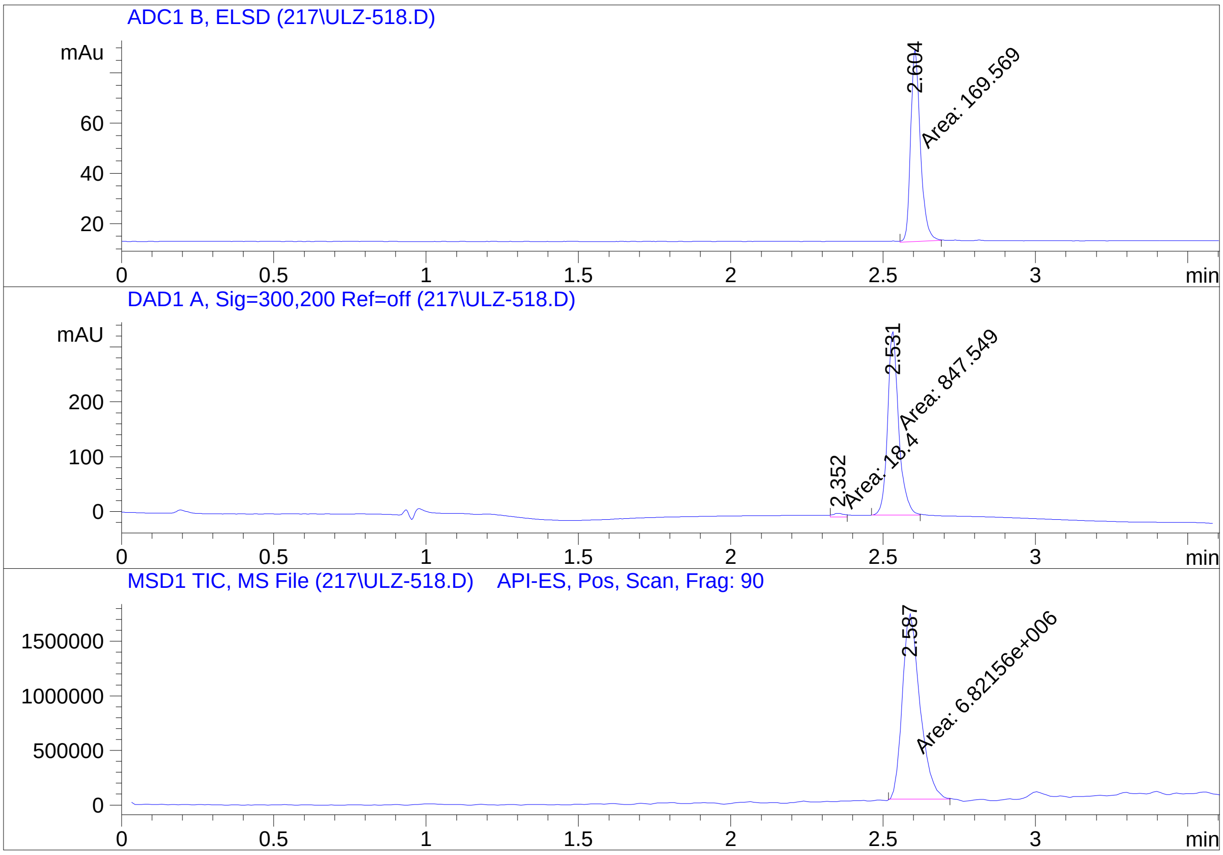

===

Area Percent Report

===

Sorted By	:	Signal
Multiplier	:	1.0000
Dilution	:	1.0000

Use Multiplier & Dilution Factor with ISTDs

Signal 1: ADC1 B, ELSD

Peak #	RetTime [min]	Type	Width [min]	Area [mAu*s]	Height [mAu]	Area %
1	2.604	MM	0.0370	169.56920	76.43751	100.0000
Totals :				169.56920	76.43751	

Signal 2: DAD1 A, Sig=300,200 Ref=off

Peak #	RetTime [min]	Type	Width [min]	Area [mAU*s]	Height [mAU]	Area %
1	2.352	MM	0.0436	18.40002	7.02679	2.1248
2	2.531	MM	0.0419	847.54889	337.29834	97.8752
Totals :				865.94891	344.32513	

Signal 3: MSD1 TIC, MS File

Peak #	RetTime [min]	Type	Width [min]	Area	Height	Area %
1	2.587	MM	0.0663	6.82156e6	1.71399e6	100.0000

Totals : 6.82156e6 1.71399e6

==

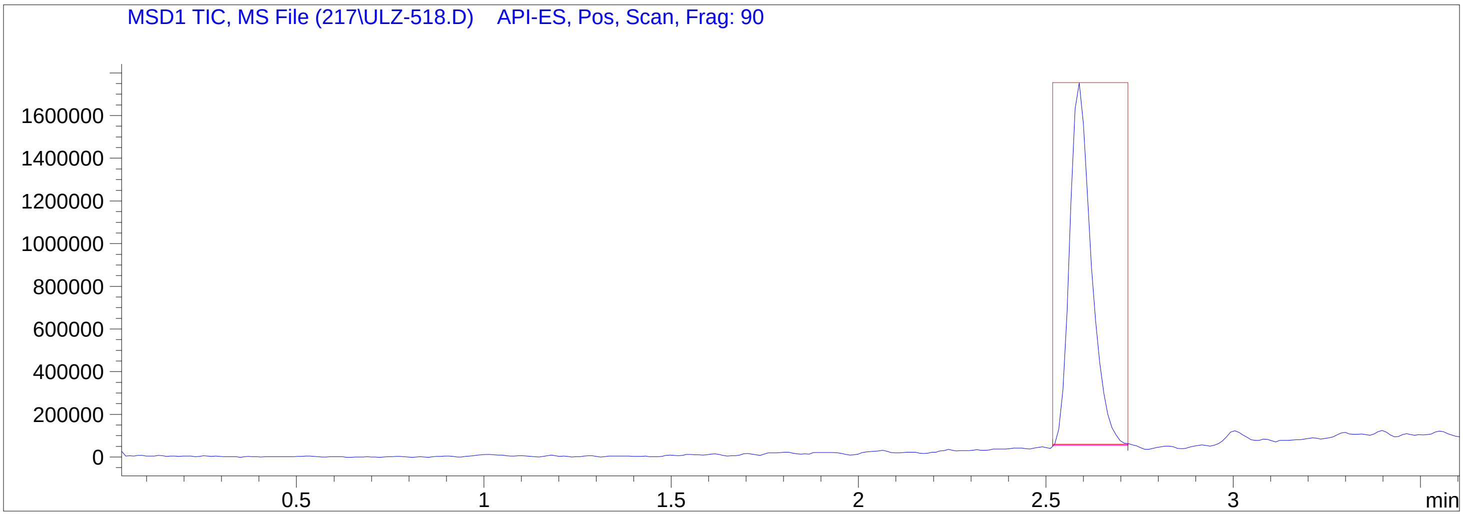

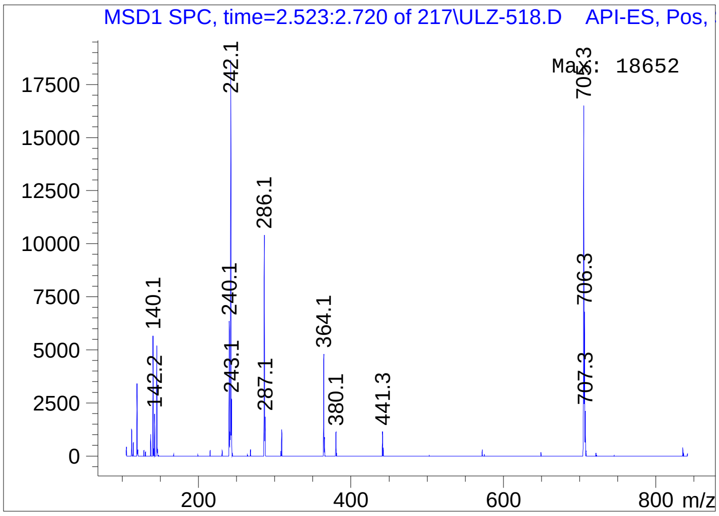

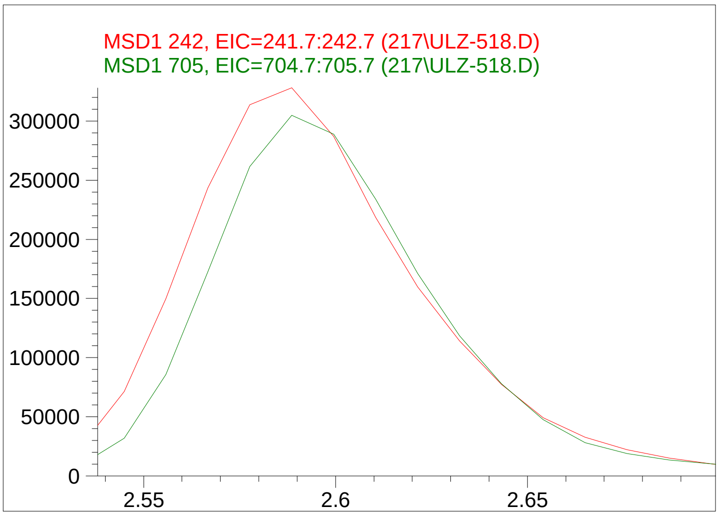


```
Peak #1 at  2.587 min ( 2.518 to  2.719 min)
-> The analysis found only one component, indicating a pure peak. <-

Component 1:  Peak at Scan 234.9. Top ions are  242 705 286
```

*** End of Report ***
