# Supplementary material for: 3-(3-Azabicyclo[2, 2, 1]heptan-2-yl)-1,2,4-oxadiazoles as Novel Potent DPP-4 Inhibitors to Treat T2DM
Source: Pharmaceuticals (Basel). 2025 Apr 28;18(5):642. doi: 10.3390/ph18050642 (PMC12114571; doi:10.3390/ph18050642)
Supplement: Supplementary file 1 [file pharmaceuticals-18-00642-s001.zip › LCMS/9a_LCMS.pdf]

```
=====================================================================
Injection Date  : 29/3/23 10:08:13 PM            Seq. Line :  11
Sample Name     : ULZ-524                         Location : P1-E-07
Acq. Operator   : #6                                   Inj :   1
Acq. Instrument : Instrument 1                  Inj Volume : Inj prog
Method          : C:\HPCHEM\1\METHODS\1PH08.M
Last changed    : 20/3/23 08:15:00 PM by #6
Column: Onyx C18 50x2.1mm | 0.80ml/min | Columns Reg Valve
Gradient: "A"->@2.0min->"B"(Hold 0.6min)->@0.05min->"A"(Hold 0.95min)->PostRun
=====================================================================

Instrument Conditions   :    At Start          At Stop
Pressure                :     116.8              71.2   bar
Flow                    :       0.800             0.800 ml/min

Detector Lamp Burn Times: Current On-Time  Accumulated On-Time
DAD 1, UV Lamp          :       0.94          86640.7   h
DAD 1, Visible Lamp     :      OFF            13251.8   h

Solvent Description     :
PMP1, Solvent A         : 0.1%TFA in Acn/H2O (2.5:97.5)
PMP1, Solvent B         : 0.1%TFA in AcN
PMP1, Solvent C         : 0.1%FA in Acn/H2O (2.5:97.5)
PMP1, Solvent D         : 0.1%FA in AcN
=====================================================================
MSD parameters
Tune file name              :    C:\HPCHEM\1\1956ATUN\atunes.tun
Ionization mode             :    APCI

MSD Instrument Conditions   :      At Start        At Stop
Quad Temp                   :            99             99 C
Gas Temp                    :           350            350 C
Vaporizer                   :           324            329 C
RoughVac                    :             2              2 Torr
HighVac                     :      1.2E-005       1.2E-005 Torr
CapCur                      :            86            792 nA
ChamCur                     :             4              0 µA
CoronaVol                   :          2784             39 Volt
DryingGas                   :             4              4 l/min
Neb Pres                    :            50             50 psig
Turbo1Spd                   :            99             99 %
Turbo1Pwr                   :            95             96 W
RF Drive                    :      0.0E-001       0.0E-001 %
Qd TpDrv                    :             9              9 %
Gas TpDrv                   :            15             12 %
Vap TpDrv                   :            34             37 %
Neb PrDrv                   :            42             42 %
Gas FlDrv                   :             5              5 %
DelaySens                   :      0.0E-001       0.0E-001 V
Aux Input                   :      0.0E-001       0.0E-001 V
Other Det                   :      0.0E-001       0.0E-001 V
=====================================================================
MSD tuning (calibration) parameters

Ionization polarity         :    Positive
Skim1                       :    Not Applicable
Skim2                       :    8.0 V
Ion Energy                  :    5.0 V
Lens1                       :    3.1 V
Lens2                       :    36 V
Iris                        :    -200 V
HED                         :    10000 V
Width Gain                  :    -893
Width Offset                :    Variable
                                 Mass         :   Value
                                 -------------------------
                                 121.05       :   -154
                                 622.03       :   -154
                                 922.01       :   -154
                                 -------------------------
Mass Gain                   :    -36.15
Mass Offset                 :    Variable
                                 Mass         :   Value
                                 -------------------------
```

```
                                 121.05       :   0.624
                                 622.03       :   0.672
                                 922.01       :   0.624
                                 -------------------------
Quad DC                         :    0.00 V
Octopole Peak                   :    650 V
Octopole Knee                   :    Not Applicable
Lens2DC                         :    Not Applicable
L2RFEn                          :    Not Applicable
L2RFPh                          :    Not Applicable
L2RFAmp                         :    Not Applicable
Mass Filter                     :    Gaussian
Mass Filter Width               :    0.30 Da
Time Filter                     :    Gaussian
Time Filter Width               :    0.030 minutes
```

===

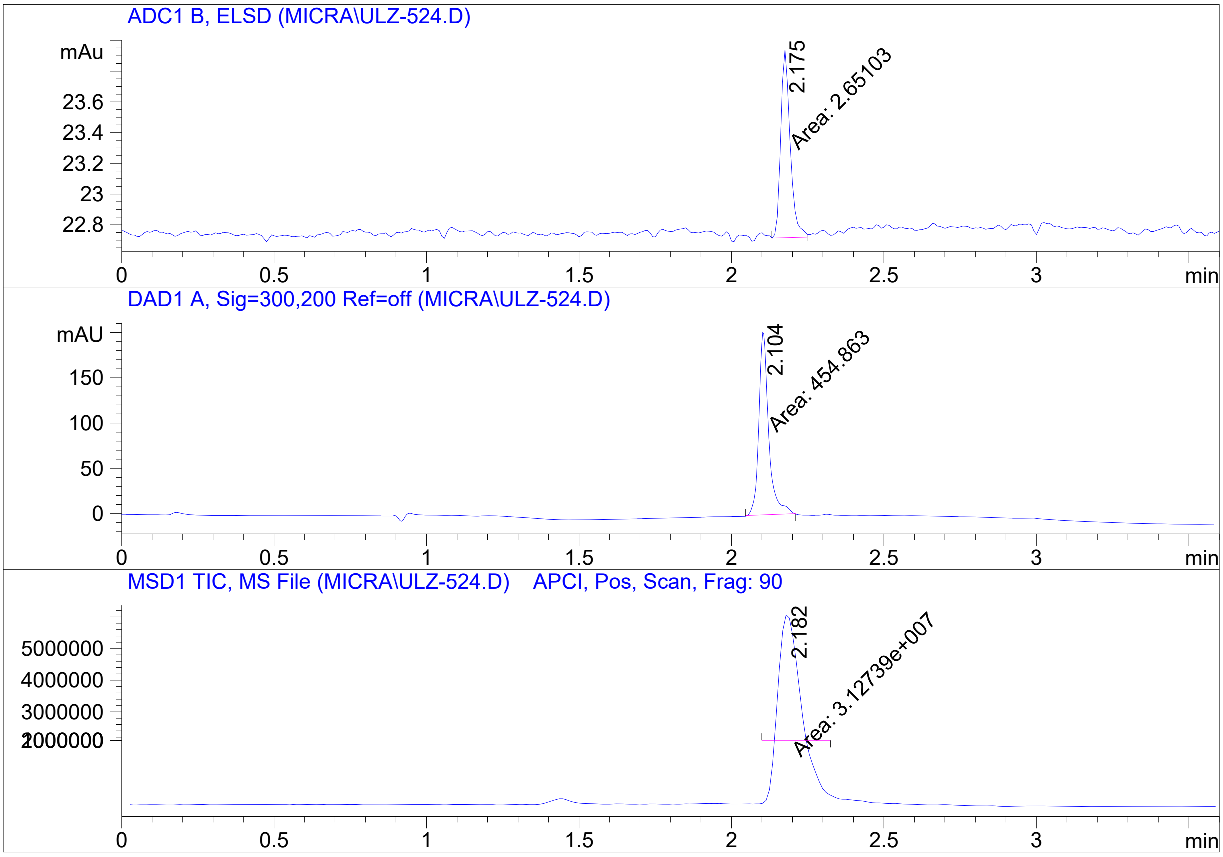

===

Area Percent Report

===

```
Sorted By              :      Signal
Multiplier             :      1.0000
Dilution               :      1.0000
Use Multiplier & Dilution Factor with ISTDs
```

Signal 1: ADC1 B, ELSD

Peak #	RetTime [min]	Type	Width [min]	Area [mAu*s]	Height [mAu]	Area %
1	2.175	MM	0.0356	2.65103	1.24146	100.0000

Totals : 2.65103   1.24146

Signal 2: DAD1 A, Sig=300,200 Ref=off

Peak #	RetTime [min]	Type	Width [min]	Area [mAU*s]	Height [mAU]	Area %
1	2.104	MM	0.0370	454.86328	205.09325	100.0000
Totals :				454.86328	205.09325	

Signal 3: MSD1 TIC, MS File

Peak #	RetTime [min]	Type	Width [min]	Area	Height	Area %
1	2.182	MM	0.0873	3.12739e7	5.96991e6	100.0000
Totals :				3.12739e7	5.96991e6	

==

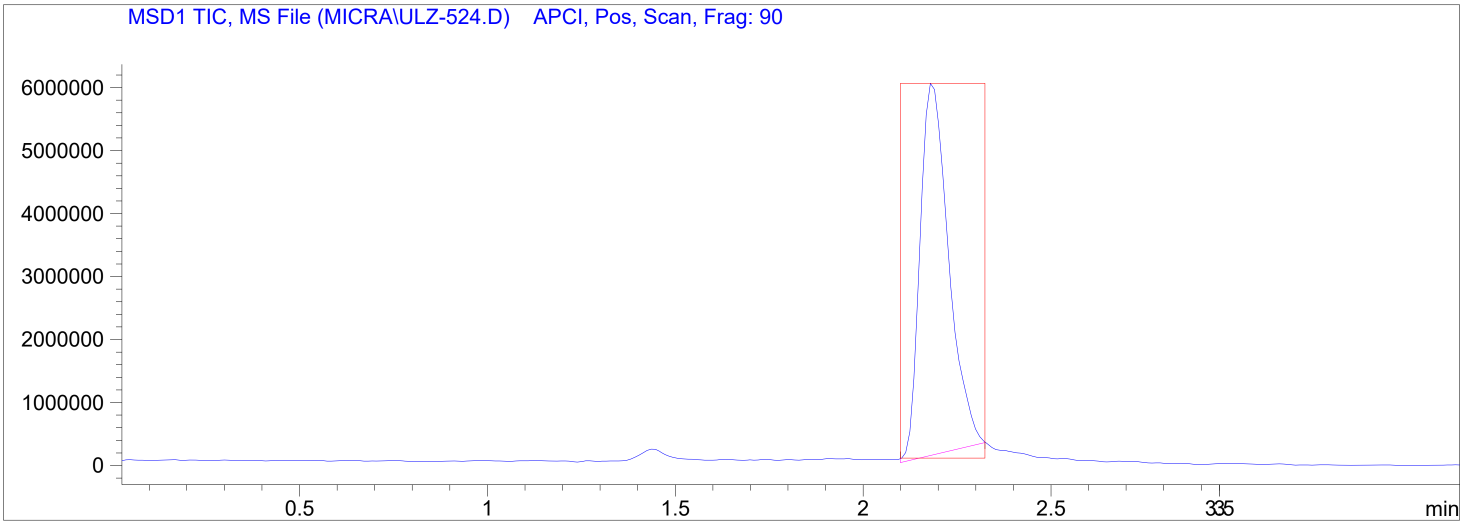

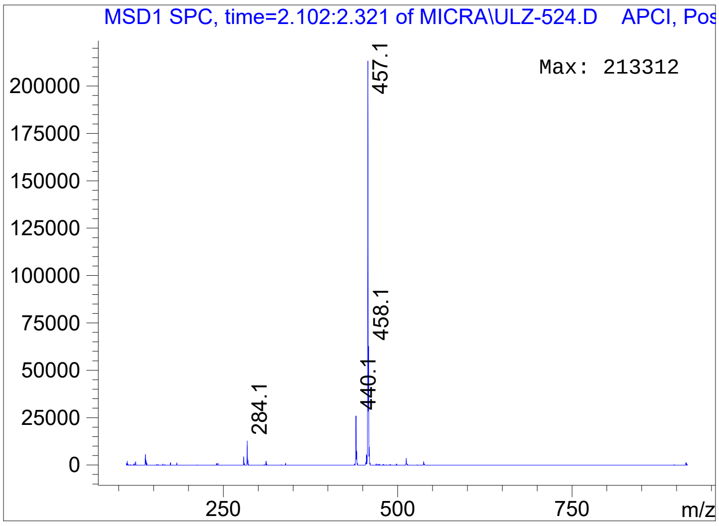

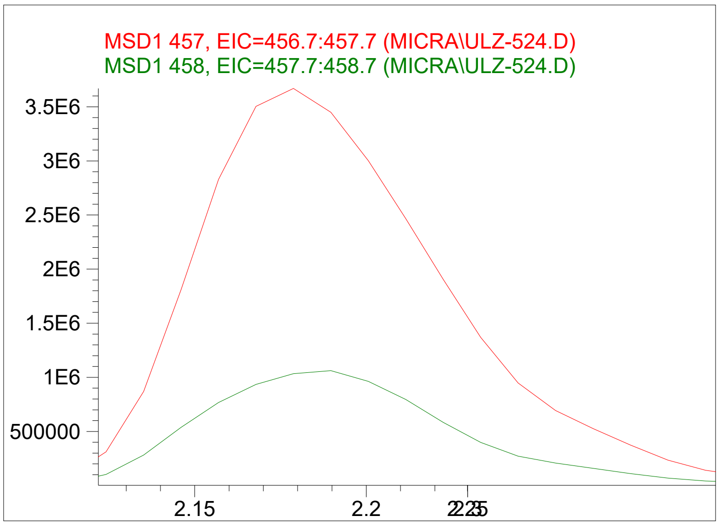


```
Peak #1 at  2.182 min ( 2.099 to  2.324 min)
-> The analysis found 2 components, indicating an impure peak. <-

Component 1:  Peak at Scan 197.9. Top ions are  457
Component 2:  Peak at Scan 198.7. Top ions are  458
```

*** End of Report ***
