# Supplementary material for: 3-(3-Azabicyclo[2, 2, 1]heptan-2-yl)-1,2,4-oxadiazoles as Novel Potent DPP-4 Inhibitors to Treat T2DM
Source: Pharmaceuticals (Basel). 2025 Apr 28;18(5):642. doi: 10.3390/ph18050642 (PMC12114571; doi:10.3390/ph18050642)
Supplement: Supplementary file 1 [file pharmaceuticals-18-00642-s001.zip › NMR/2b_NMR/2b_COSY alifatic region.pdf]

ULZ-534 DMSO-D6 Dsh 23_05_02-1/03 COSY

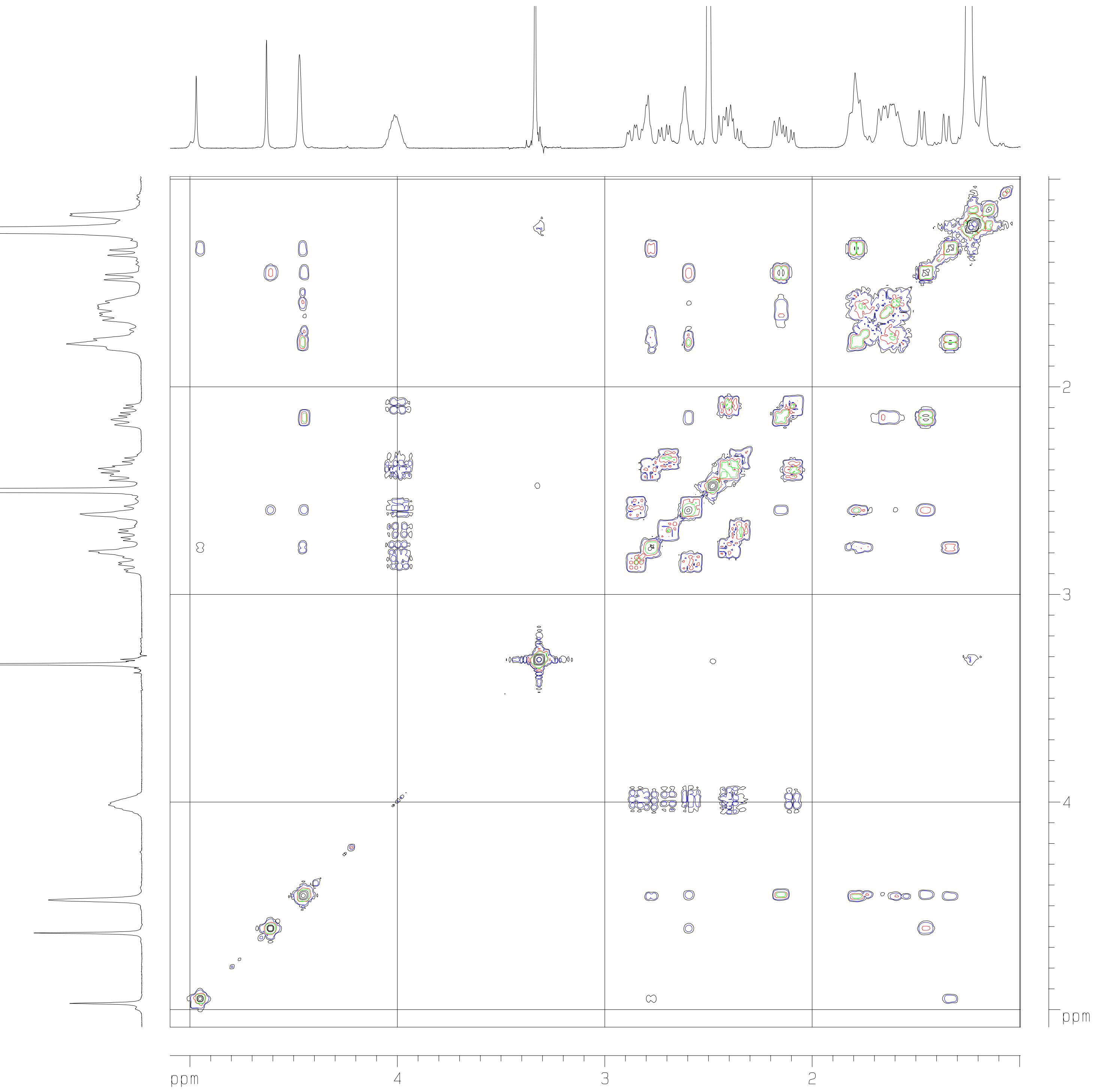

Current Data Parameters

NAME	ULZ-534
EXPNO	30
PROCNO	1

F2 - Acquisition Parameters

Date_	20230503
Time	10.24
INSTRUM	spect
PROBHD	5 mm Multinucl
PULPROG	cosygpqf
TD	1024
SOLVENT	DMSO
NS	1
DS	16
SWH	3306.878 Hz
FIDRES	3.229373 Hz
AQ	0.1548788 sec
RG	20
DW	151.200 usec
DE	6.00 usec
TE	0.0 K
d0	0.00000300 sec
D1	1.00000000 sec
d13	0.00000400 sec
D16	0.00010000 sec
IN0	0.00030256 sec
MCREST	0.00000000 sec
MCWRK	1.00000000 sec

======== CHANNEL f1 ========

NUC1	1H
P0	10.00 usec
P1	10.00 usec
PL1	0.00 dB
SFO1	400.1318850 MHz

====== GRADIENT CHANNEL =====

GPNAM1	SINE.100
GPNAM2	SINE.100
GPX1	0.00 %
GPX2	0.00 %
GPY1	0.00 %
GPY2	0.00 %
GPZ1	20.00 %
GPZ2	20.00 %
P16	1600.00 usec

F1 - Acquisition parameters

ND0	1
TD	512
SFO1	400.1319 MHz
FIDRES	6.455278 Hz
SW	8.260 ppm
FnMODE	QF

F2 - Processing parameters

SI	1024
SF	400.1300108 MHz
WDW	QSINE
SSB	0
LB	0.00 Hz
GB	0
PC	0.60

F1 - Processing parameters

SI	1024
MC2	QF
SF	400.1300120 MHz
WDW	QSINE
SSB	0
LB	0.00 Hz
GB	0

2D NMR plot parameters

CX2	15.00 cm
CX1	15.00 cm
F2PLO	5.096 ppm
F2LO	2038.91 Hz
F2PHI	0.996 ppm
F2HI	398.39 Hz
F1PLO	5.084 ppm
F1LO	2034.42 Hz
F1PHI	0.987 ppm
F1HI	394.77 Hz
F2PPMCM	0.27333 ppm/cm
F2HZCM	109.36811 Hz/cm
F1PPMCM	0.27318 ppm/cm
F1HZCM	109.30937 Hz/cm
