# Supplementary material for: 3-(3-Azabicyclo[2, 2, 1]heptan-2-yl)-1,2,4-oxadiazoles as Novel Potent DPP-4 Inhibitors to Treat T2DM
Source: Pharmaceuticals (Basel). 2025 Apr 28;18(5):642. doi: 10.3390/ph18050642 (PMC12114571; doi:10.3390/ph18050642)
Supplement: Supplementary file 1 [file pharmaceuticals-18-00642-s001.zip › NMR/3a_NMR/3a_HSQC.pdf]

ULZ-520 DMSO-D6 Dsh 23_03_28-5/01 HSQC

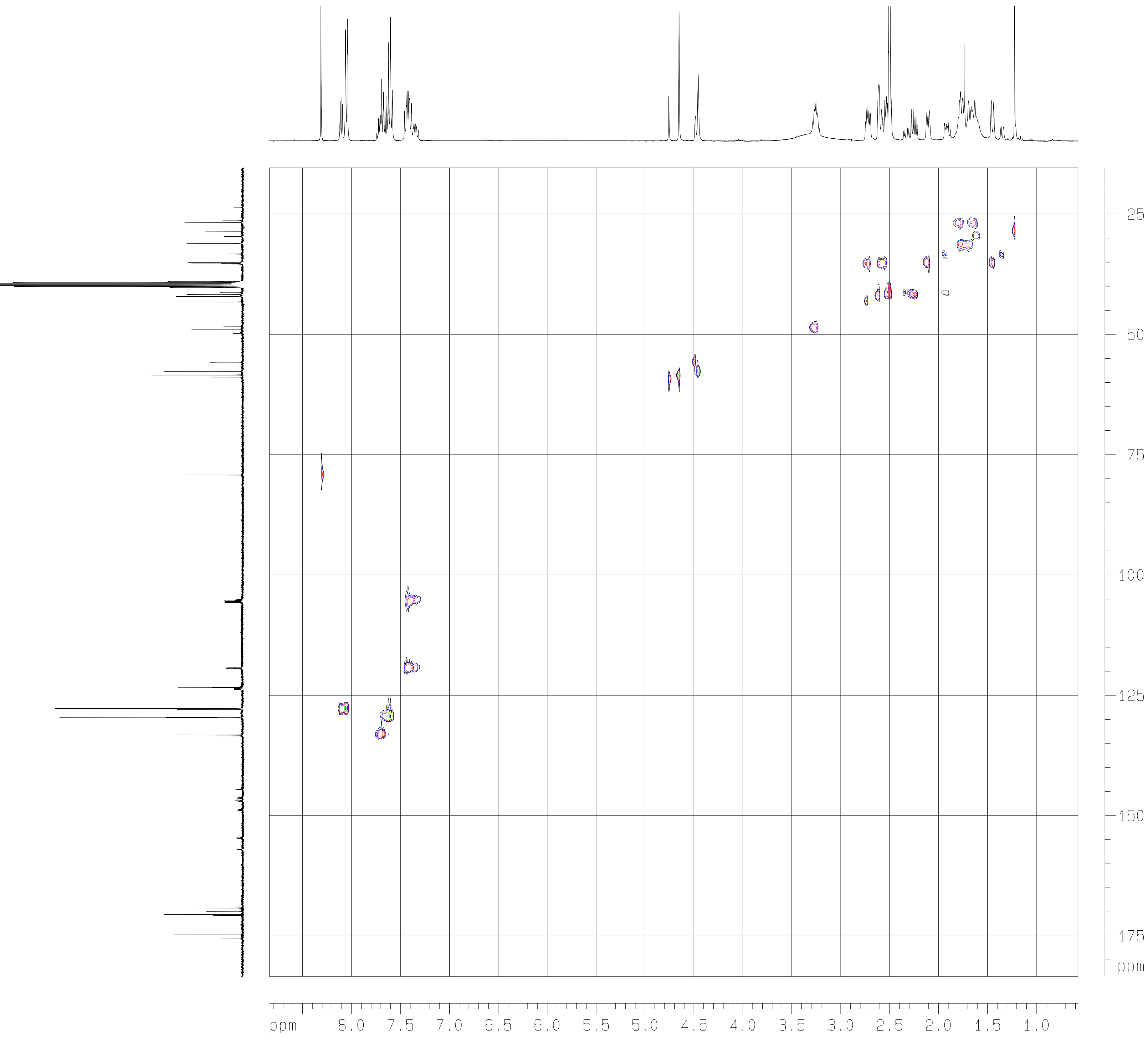

Current Data Parameters

NAME	ULZ-520
EXPNO	50
PROCNO	1

F2 - Acquisition Parameters

Date_	20230329
Time	10.11
INSTRUM	spect
PROBHD	5 mm Multinucl
PULPROG	invietgp
TD	2048
SOLVENT	DMSO
NS	4
DS	16
SWH	3306.878 Hz
FIDRES	1.614687 Hz
AQ	0.3097076 sec
RG	16384
DW	151.200 usec
DE	6.00 usec
TE	0.0 K
CNST2	180.0000000
d0	0.00000300 sec
D1	1.00000000 sec
d4	0.00138889 sec
d11	0.03000000 sec
d13	0.00000400 sec
D16	0.00015000 sec
DELTA	0.00117500 sec
DELTA1	0.00038089 sec
IN0	0.00002957 sec
MCREST	0.00000000 sec
MCWRK	0.20000000 sec
ST1CNT	128

======== CHANNEL f1 ========

NUC1	1H
P1	9.50 usec
p2	19.00 usec
P28	2000.00 usec
PL1	0.00 dB
SFO1	400.1318850 MHz

======== CHANNEL f2 ========

CPDPRG2	garp
NUC2	13C
P3	14.50 usec
p4	29.00 usec
PCPD2	80.00 usec
PL2	-6.00 dB
PL12	8.70 dB
SFO2	100.6228140 MHz

====== GRADIENT CHANNEL =====

GPNAM1	SINE.100
GPNAM2	SINE.100
GPX1	0.00 %
GPX2	0.00 %
GPY1	0.00 %
GPY2	0.00 %
GPZ1	80.00 %
GPZ2	20.10 %
P16	1000.00 usec

F1 - Acquisition parameters

ND0	2
TD	256
SFO1	100.6228 MHz
FIDRES	66.039726 Hz
SW	168.015 ppm
FnMODE	Echo-Antiecho

F2 - Processing parameters

SI	2048
SF	400.1300017 MHz
WDW	QSINE
SSB	2
LB	0.00 Hz
GB	0
PC	1.00

F1 - Processing parameters

SI	1024
MC2	echo-antiecho
SF	100.6128132 MHz
WDW	QSINE
SSB	2
LB	0.00 Hz
GB	0

2D NMR plot parameters

CX2	15.00 cm
CX1	15.00 cm
F2PLO	8.839 ppm
F2LO	3536.76 Hz
F2PHI	0.575 ppm
F2HI	229.88 Hz
F1PLO	183.415 ppm
F1LO	18453.89 Hz
F1PHI	15.383 ppm
F1HI	1547.72 Hz
F2PPMCM	0.55097 ppm/cm
F2HZCM	220.45856 Hz/cm
F1PPMCM	11.20213 ppm/cm
F1HZCM	1127.07800 Hz/cm
