# Supplementary material for: 3-(3-Azabicyclo[2, 2, 1]heptan-2-yl)-1,2,4-oxadiazoles as Novel Potent DPP-4 Inhibitors to Treat T2DM
Source: Pharmaceuticals (Basel). 2025 Apr 28;18(5):642. doi: 10.3390/ph18050642 (PMC12114571; doi:10.3390/ph18050642)
Supplement: Supplementary file 1 [file pharmaceuticals-18-00642-s001.zip › NMR/3a_NMR/3a_NMR 13C alifatic region.pdf]

ULZ-520 DMSO-D6 Dsh 23_03_28-5/01 NMR 13C

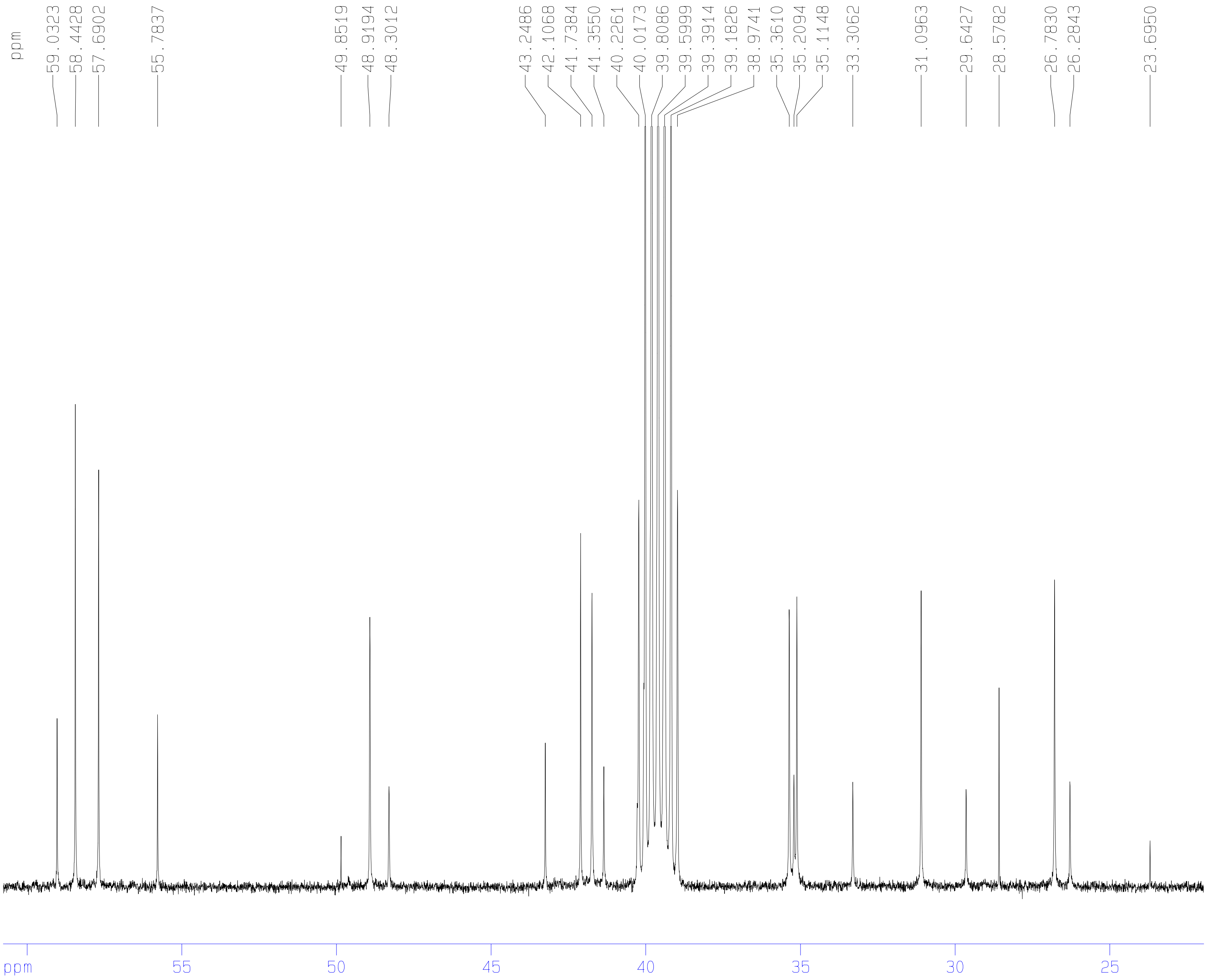

Current Data Parameters

NAME	ULZ-520
EXPNO	13
PROCNO	1

F2 - Acquisition Parameters

Date_	20230328
Time	19.04
INSTRUM	spect
PROBHD	5 mm Multinucl
PULPROG	zgpg
TD	32768
SOLVENT	DMSO
NS	24576
DS	0
SWH	19083.969 Hz
FIDRES	0.582397 Hz
AQ	0.8585716 sec
RG	16384
DW	26.200 usec
DE	6.00 usec
TE	0.0 K
D1	1.00000000 sec
d11	0.03000000 sec
DELTA	0.89999998 sec
MCREST	0.00000000 sec
MCWRK	0.01500000 sec

======== CHANNEL f1 ========

NUC1	13C
P1	7.00 usec
PL1	-3.00 dB
SFO1	100.6223272 MHz

======== CHANNEL f2 ========

CPDPRG2	waltz16
NUC2	1H
PCPD2	100.00 usec
PL2	-2.00 dB
PL12	18.00 dB
PL13	18.00 dB
SFO2	400.1320000 MHz

F2 - Processing parameters

SI	32768
SF	100.6128102 MHz
WDW	EM
SSB	0
LB	0.10 Hz
GB	0
PC	0.80

1D NMR plot parameters

CX	21.00 cm
CY	50.00 cm
F1P	60.774 ppm
F1	6114.66 Hz
F2P	21.961 ppm
F2	2209.52 Hz
PPMCM	1.84827 ppm/cm
HZCM	185.95930 Hz/cm
