# Supplementary material for: 3-(3-Azabicyclo[2, 2, 1]heptan-2-yl)-1,2,4-oxadiazoles as Novel Potent DPP-4 Inhibitors to Treat T2DM
Source: Pharmaceuticals (Basel). 2025 Apr 28;18(5):642. doi: 10.3390/ph18050642 (PMC12114571; doi:10.3390/ph18050642)
Supplement: Supplementary file 1 [file pharmaceuticals-18-00642-s001.zip › NMR/3b_NMR/3b_HSQC alifatic region.pdf]

ULZ-555-1 DMSO-D6 Dsh 23_07_10-2/03 HSQC

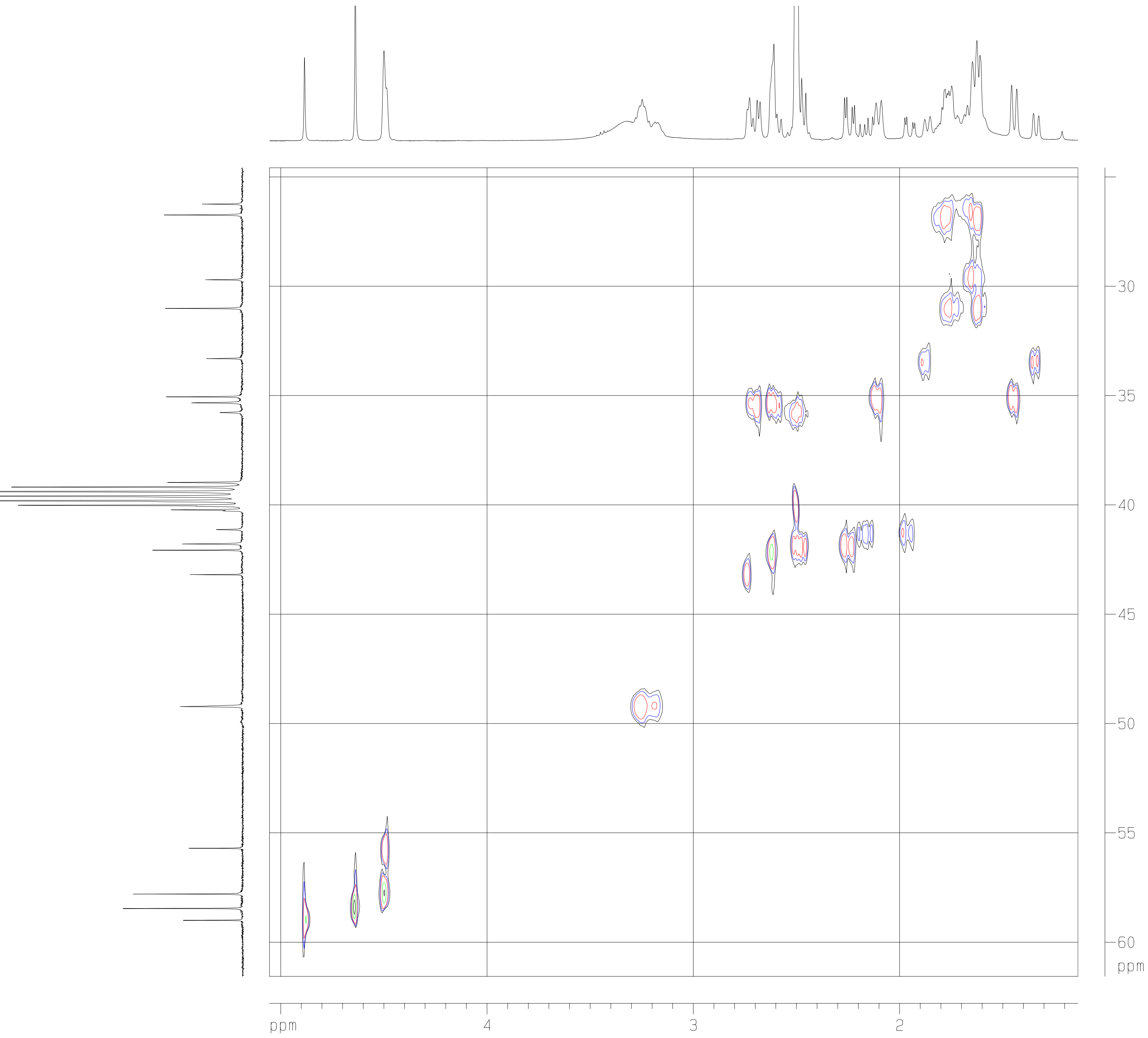

Current Data Parameters

NAME	ULZ-555-1	
EXPNO	50	
PROCNO	1	

F2 - Acquisition Parameters

Date_	20230711	
Time	12.41	
INSTRUM	spect	
PROBHD	5 mm Multinucl	
PULPROG	invietgp	
TD	2048	
SOLVENT	DMSO	
NS	2	
DS	16	
SWH	3930.818	Hz
FIDRES	1.919345	Hz
AQ	0.2605556	sec
RG	16384	
DW	127.200	usec
DE	6.00	usec
TE	0.0	K
CNST2	180.0000000	
d0	0.00000300	sec
D1	1.00000000	sec
d4	0.00138889	sec
d11	0.03000000	sec
d13	0.00000400	sec
D16	0.00015000	sec
DELTA	0.00117500	sec
DELTA1	0.00038089	sec
IN0	0.00004107	sec
MCREST	0.00000000	sec
MCWRK	0.20000000	sec
ST1CNT	128	

======== CHANNEL f1 ========

NUC1	1H	
P1	9.50	usec
p2	19.00	usec
P28	2000.00	usec
PL1	0.00	dB
SFO1	400.1318590	MHz

======== CHANNEL f2 ========

CPDPRG2	garp	
NUC2	13C	
P3	14.50	usec
p4	29.00	usec
PCPD2	80.00	usec
PL2	-6.00	dB
PL12	8.70	dB
SFO2	100.6208380	MHz

====== GRADIENT CHANNEL =====

GPNAM1	SINE.100	
GPNAM2	SINE.100	
GPX1	0.00	%
GPX2	0.00	%
GPY1	0.00	%
GPY2	0.00	%
GPZ1	80.00	%
GPZ2	20.10	%
P16	1000.00	usec

F1 - Acquisition parameters

ND0	2	
TD	256	
SFO1	100.6208	MHz
FIDRES	47.550213	Hz
SW	120.977	ppm
FnMODE	Echo-Antiecho	

F2 - Processing parameters

SI	2048	
SF	400.1300017	MHz
WDW	QSINE	
SSB	2	
LB	0.00	Hz
GB	0	
PC	1.40	

F1 - Processing parameters

SI	1024	
MC2	echo-antiecho	
SF	100.6128132	MHz
WDW	QSINE	
SSB	2	
LB	0.00	Hz
GB	0	

2D NMR plot parameters

CX2	15.00	cm
CX1	15.00	cm
F2PLO	5.054	ppm
F2LO	2022.39	Hz
F2PHI	1.135	ppm
F2HI	454.28	Hz
F1PLO	61.564	ppm
F1LO	6194.12	Hz
F1PHI	24.583	ppm
F1HI	2473.31	Hz
F2PPMCM	0.26127	ppm/cm
F2HZCM	104.54030	Hz/cm
F1PPMCM	2.46543	ppm/cm
F1HZCM	248.05363	Hz/cm
