# Supplementary material for: 3-(3-Azabicyclo[2, 2, 1]heptan-2-yl)-1,2,4-oxadiazoles as Novel Potent DPP-4 Inhibitors to Treat T2DM
Source: Pharmaceuticals (Basel). 2025 Apr 28;18(5):642. doi: 10.3390/ph18050642 (PMC12114571; doi:10.3390/ph18050642)
Supplement: Supplementary file 1 [file pharmaceuticals-18-00642-s001.zip › NMR/3b_NMR/3b_NMR 13C alifatic region.pdf]

ULZ-555-1 DMSO-D6 Dsh 23_07_10-2/03 NMR 13C

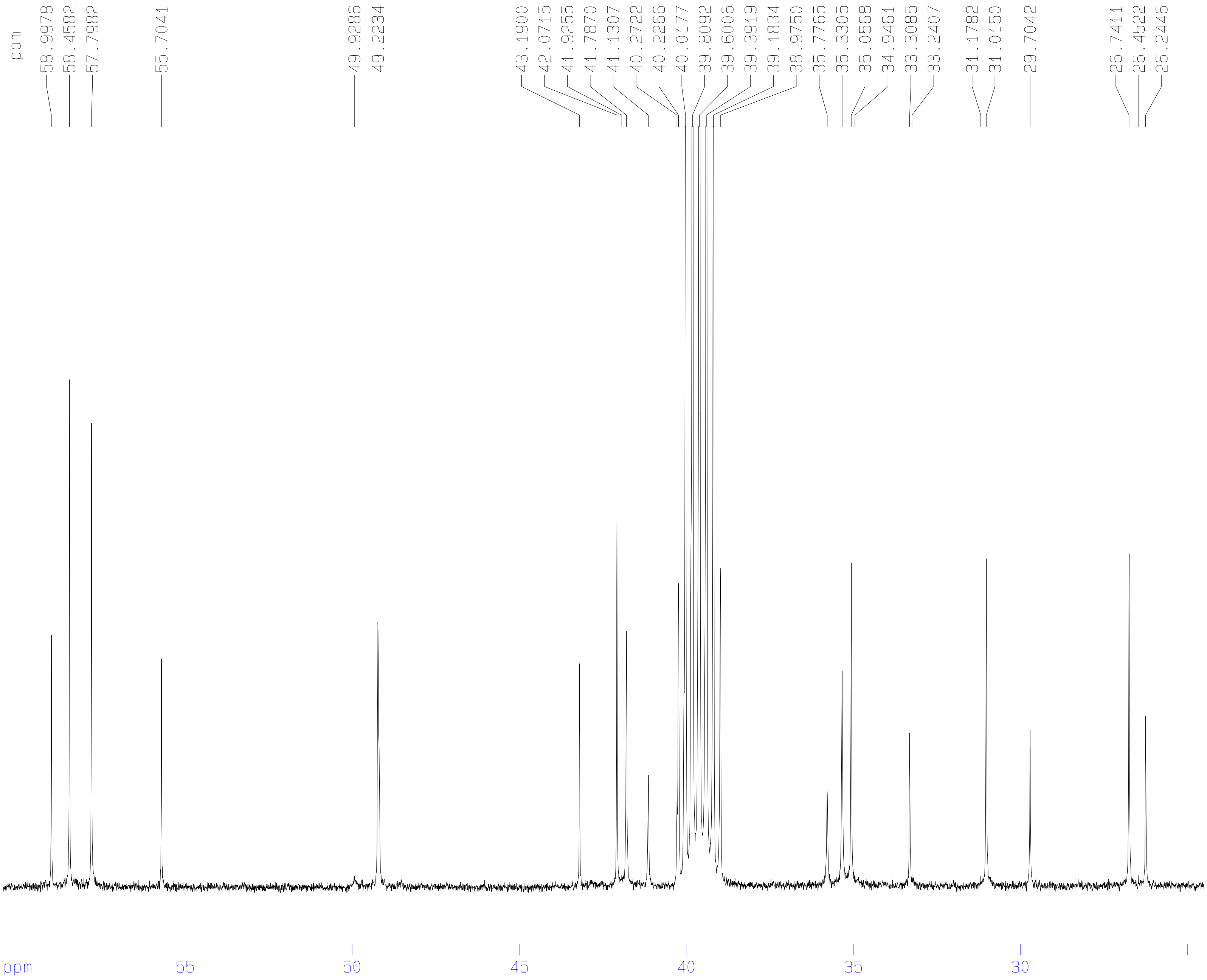

Current Data Parameters

NAME	ULZ-555-1
EXPNO	14
PROCNO	1

F2 - Acquisition Parameters

Date_	20230710
Time	17.59
INSTRUM	spect
PROBHD	5 mm Multinucl
PULPROG	zgpg
TD	32768
SOLVENT	DMSO
NS	32768
DS	0
SWH	20161.291 Hz
FIDRES	0.615274 Hz
AQ	0.8126964 sec
RG	16384
DW	24.800 usec
DE	6.00 usec
TE	0.0 K
D1	0.50000000 sec
d11	0.03000000 sec
DELTA	0.40000001 sec
MCREST	0.00000000 sec
MCWRK	0.01500000 sec

======== CHANNEL f1 ========

NUC1	13C
P1	7.00 usec
PL1	-3.00 dB
SFO1	100.6228303 MHz

======== CHANNEL f2 ========

CPDPRG2	waltz16
NUC2	1H
PCPD2	100.00 usec
PL2	-2.00 dB
PL12	18.00 dB
PL13	18.00 dB
SFO2	400.1320000 MHz

F2 - Processing parameters

SI	32768
SF	100.6128132 MHz
WDW	EM
SSB	0
LB	0.20 Hz
GB	0
PC	0.80

1D NMR plot parameters

CX	21.00 cm
CY	40.00 cm
F1P	60.441 ppm
F1	6081.16 Hz
F2P	24.508 ppm
F2	2465.77 Hz
PPMCM	1.71113 ppm/cm
HZCM	172.16129 Hz/cm
