# Supplementary material for: 3-(3-Azabicyclo[2, 2, 1]heptan-2-yl)-1,2,4-oxadiazoles as Novel Potent DPP-4 Inhibitors to Treat T2DM
Source: Pharmaceuticals (Basel). 2025 Apr 28;18(5):642. doi: 10.3390/ph18050642 (PMC12114571; doi:10.3390/ph18050642)
Supplement: Supplementary file 1 [file pharmaceuticals-18-00642-s001.zip › NMR/3b_NMR/3b_NOESY alifatic region.pdf]

ULZ-555-1 DMSO-D6 Dsh 23_07_10-2/03 NOESY d1=2 sec d8=300 msec

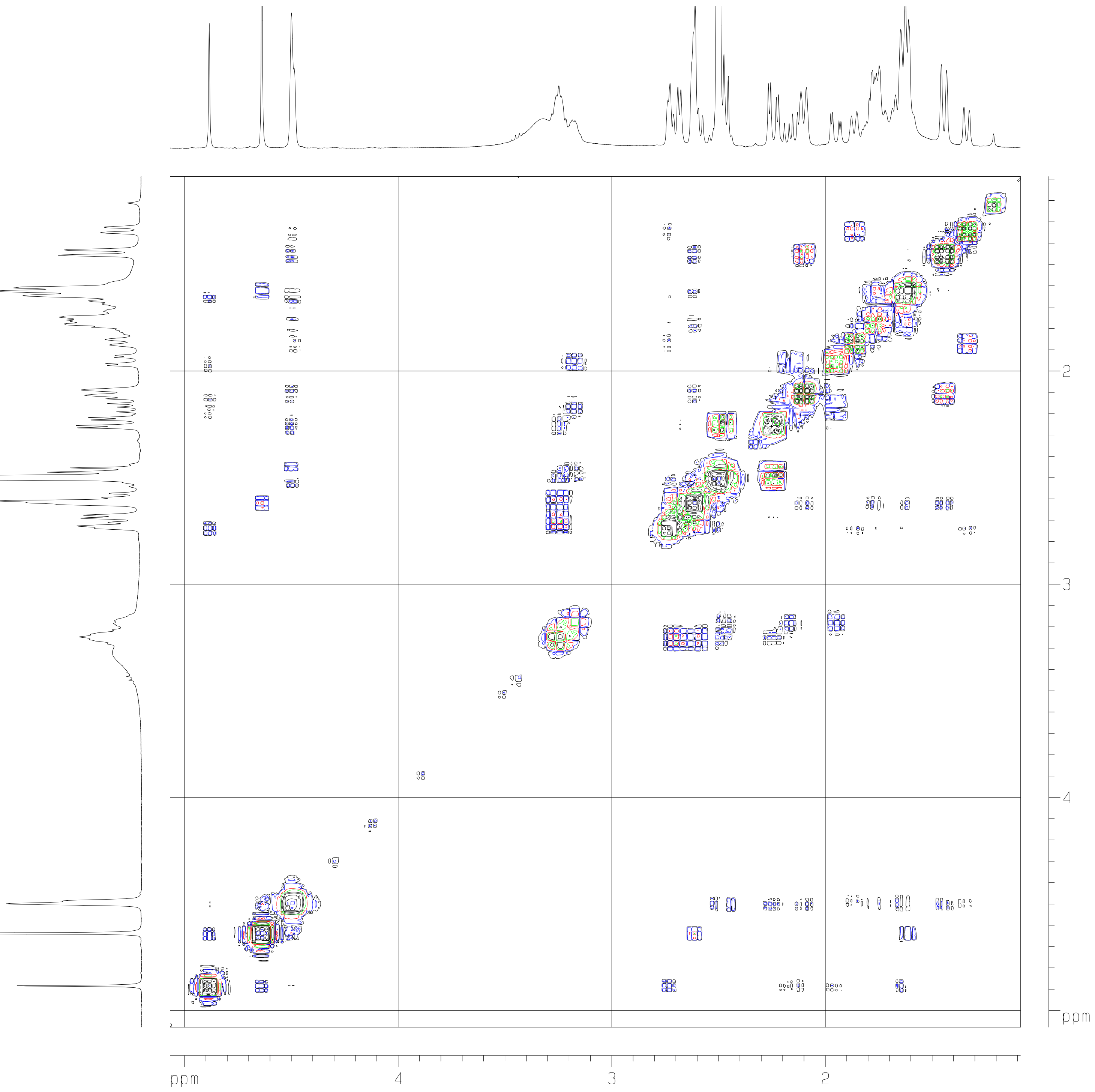

Current Data Parameters

NAME	ULZ-555-1	
EXPNO	70	
PROCNO	1	

F2 - Acquisition Parameters

Date_	20230711	
Time	12.18	
INSTRUM	spect	
PROBHD	5 mm Multinucl	
PULPROG	noesygpph	
TD	1024	
SOLVENT	DMSO	
NS	2	
DS	16	
SWH	3930.818	Hz
FIDRES	3.838689	Hz
AQ	0.1303028	sec
RG	50	
DW	127.200	usec
DE	6.00	usec
TE	0.0	K
d0	0.00011541	sec
D1	2.00000000	sec
D8	0.30000001	sec
D16	0.00100000	sec
IN0	0.00025501	sec
MCREST	0.00000000	sec
MCWRK	2.00000000	sec
TAU	0.14800000	sec

======== CHANNEL f1 ========

NUC1	1H	
P1	9.50	usec
P2	19.00	usec
PL1	0.00	dB
SFO1	400.1318590	MHz

====== GRADIENT CHANNEL =====

GPNAM1	SINE.100	
GPNAM2	SINE.100	
GPX1	0.00	%
GPX2	0.00	%
GPY1	0.00	%
GPY2	0.00	%
GPZ1	40.00	%
GPZ2	-40.00	%
P16	1000.00	usec

F1 - Acquisition parameters

ND0	1	
TD	256	
SFO1	400.1319	MHz
FIDRES	15.317877	Hz
SW	9.800	ppm
FnMODE	TPPI	

F2 - Processing parameters

SI	2048	
SF	400.1300020	MHz
WDW	QSINE	
SSB	0	
LB	0.00	Hz
GB	0	
PC	0.60	

F1 - Processing parameters

SI	2048	
MC2	TPPI	
SF	400.1300013	MHz
WDW	QSINE	
SSB	0	
LB	0.00	Hz
GB	0	

2D NMR plot parameters

CX2	15.00	cm
CX1	15.00	cm
F2PLO	5.068	ppm
F2LO	2027.85	Hz
F2PHI	1.087	ppm
F2HI	434.80	Hz
F1PLO	5.078	ppm
F1LO	2031.93	Hz
F1PHI	1.087	ppm
F1HI	435.04	Hz
F2PPMCM	0.26542	ppm/cm
F2HZCM	106.20374	Hz/cm
F1PPMCM	0.26606	ppm/cm
F1HZCM	106.45924	Hz/cm
