# Supplementary figures and images for: 3-(3-Azabicyclo[2, 2, 1]heptan-2-yl)-1,2,4-oxadiazoles as Novel Potent DPP-4 Inhibitors to Treat T2DM
Source: Pharmaceuticals (Basel). 2025 Apr 28;18(5):642. doi: 10.3390/ph18050642 (PMC12114571; doi:10.3390/ph18050642)

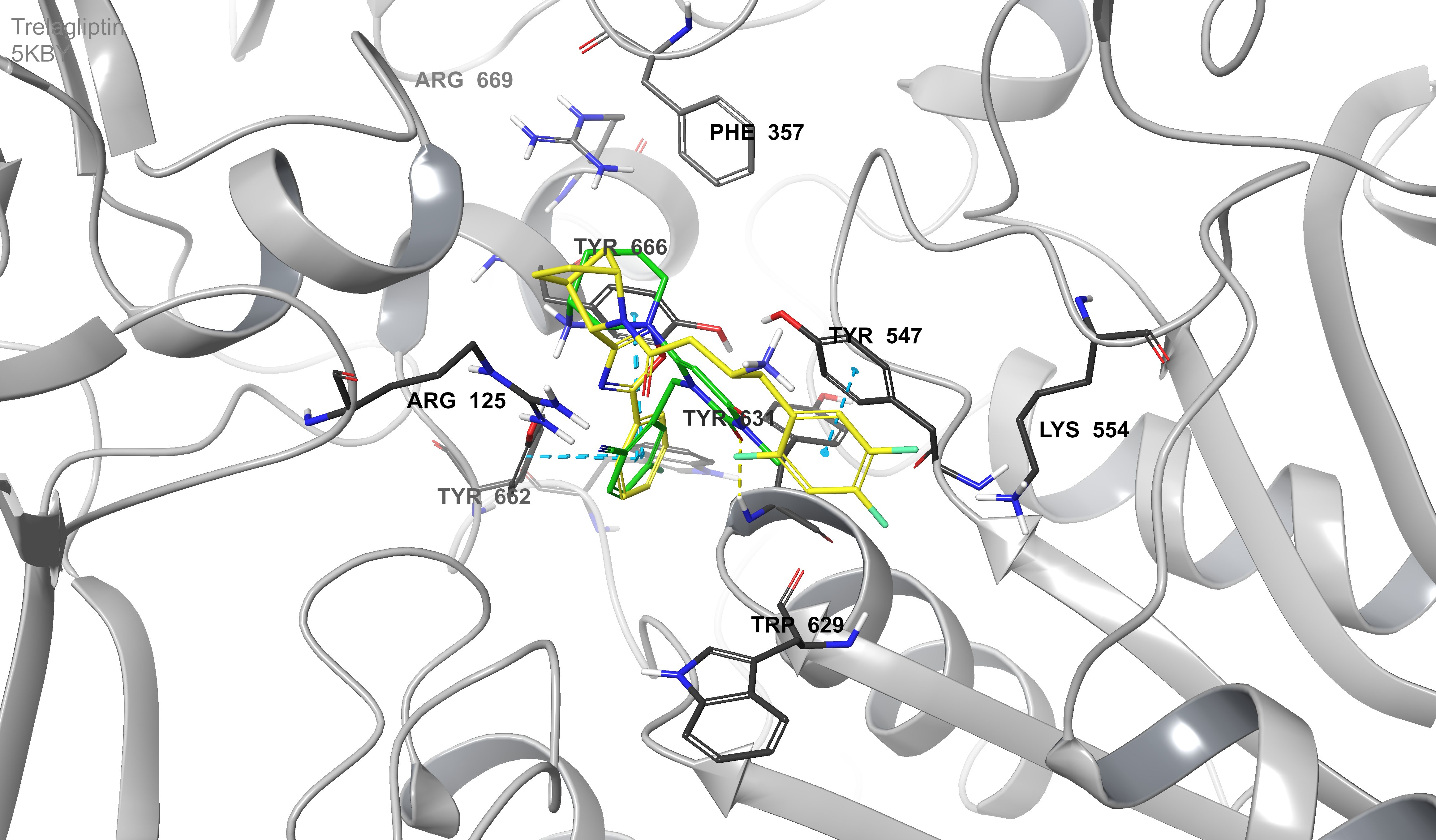

Supplement: Supplementary file 1 [file pharmaceuticals-18-00642-s001.zip › Docking images/Fig4-3a+trelagliptin.png]

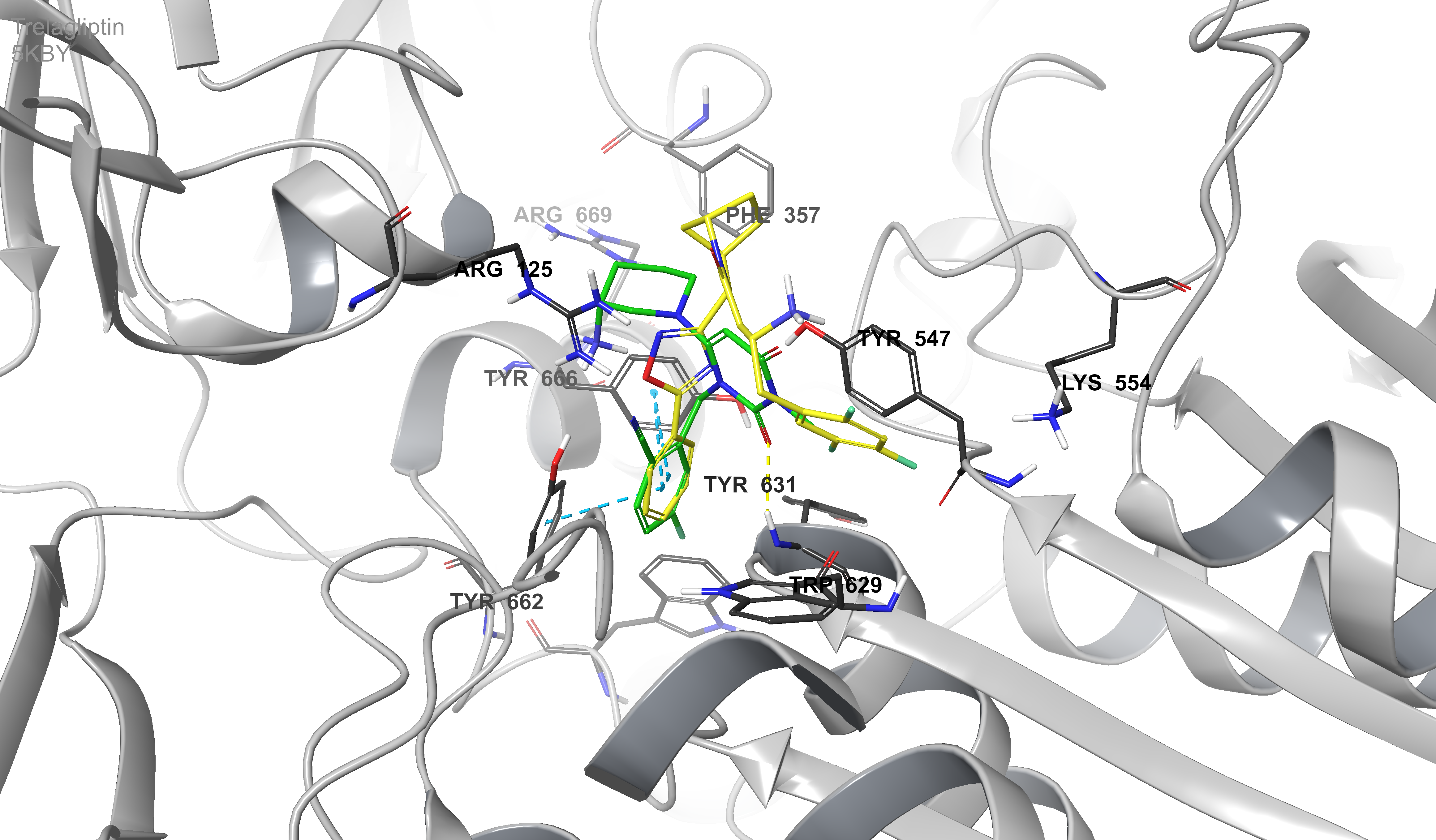

Supplement: Supplementary file 1 [file pharmaceuticals-18-00642-s001.zip › Docking images/Fig4-3b+trelagliptin.png]

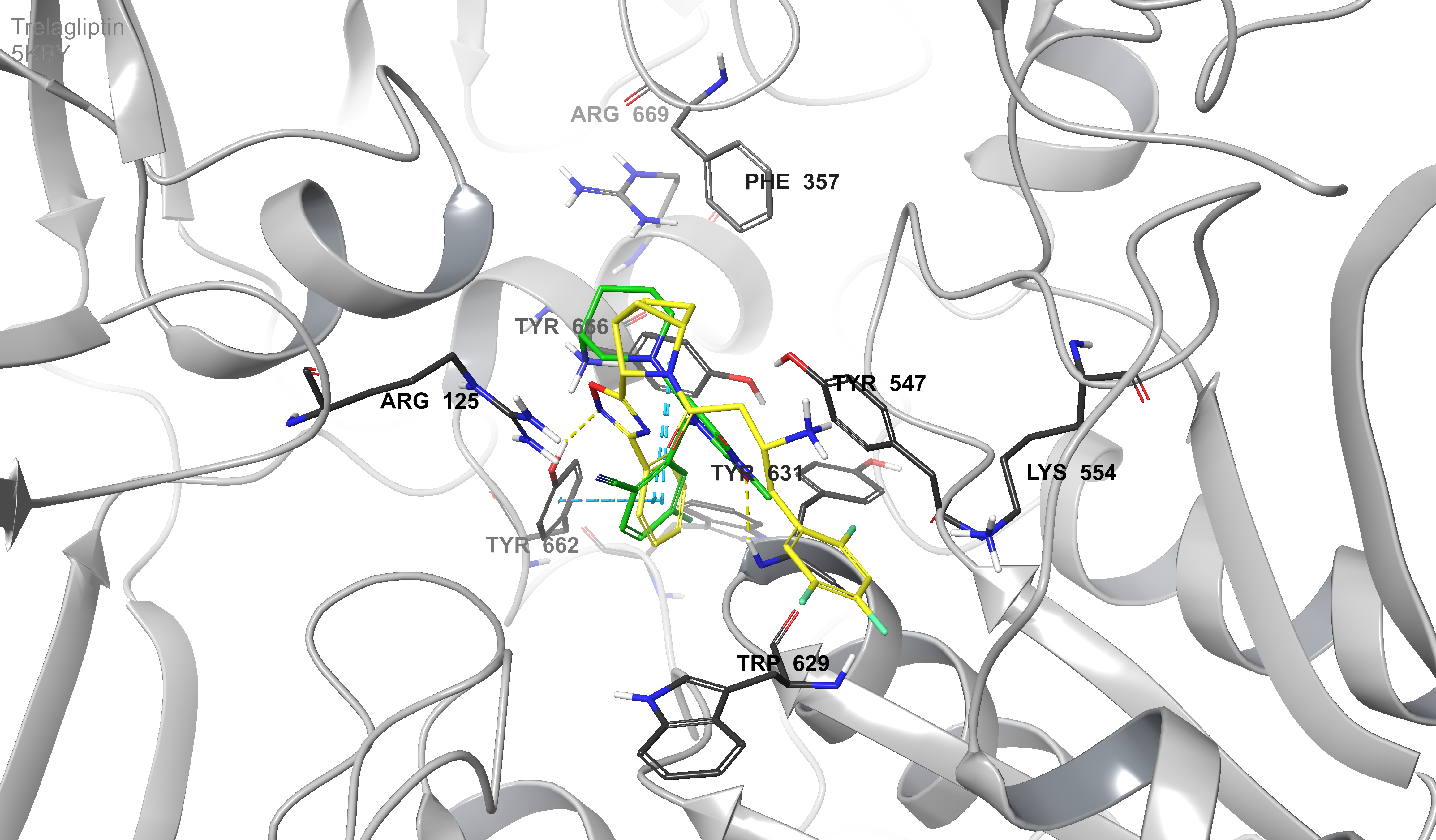

Supplement: Supplementary file 1 [file pharmaceuticals-18-00642-s001.zip › Docking images/Fig4-9a+trelagliptin.png]

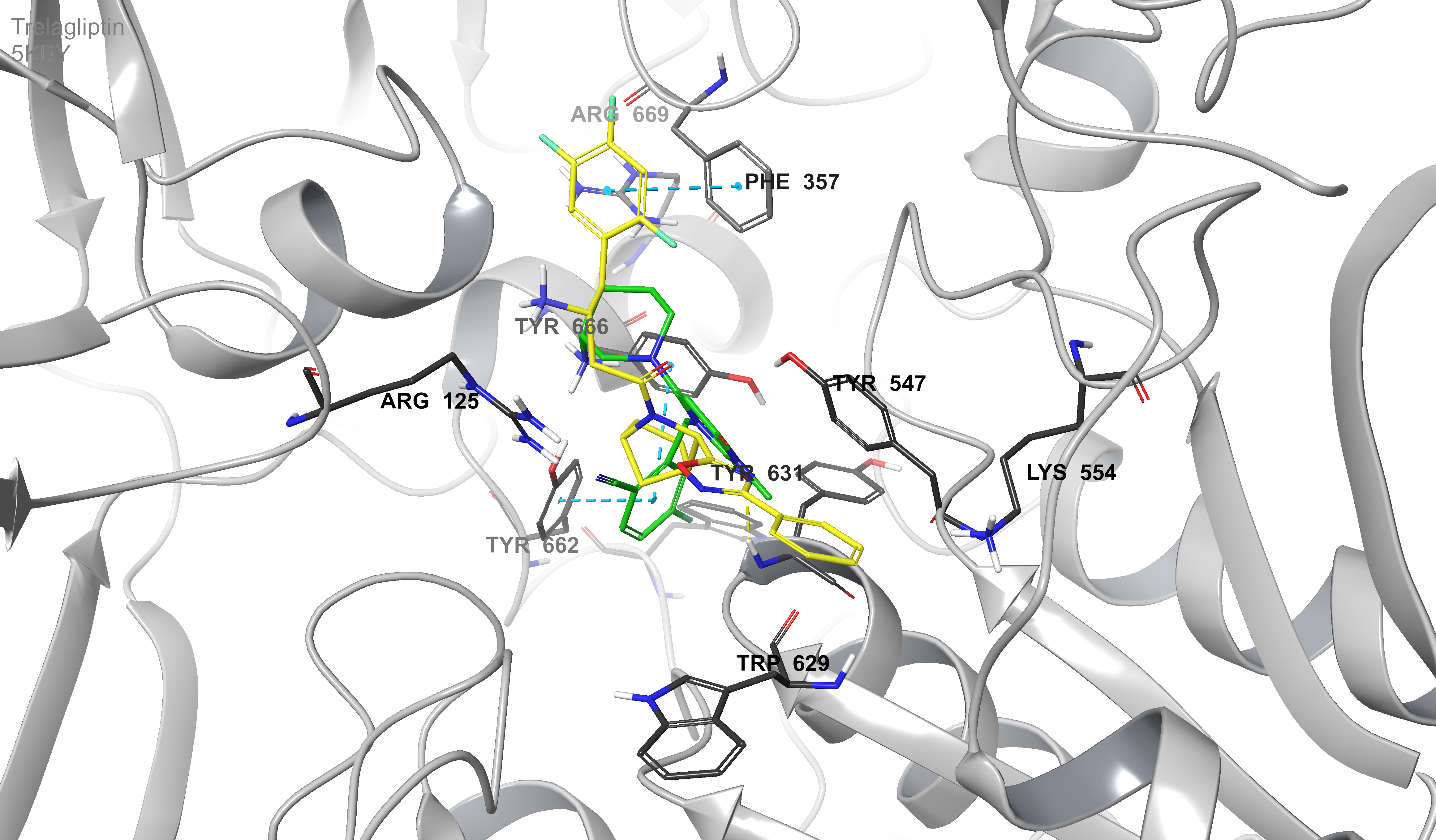

Supplement: Supplementary file 1 [file pharmaceuticals-18-00642-s001.zip › Docking images/Fig4-9b+trelagliptin.png]

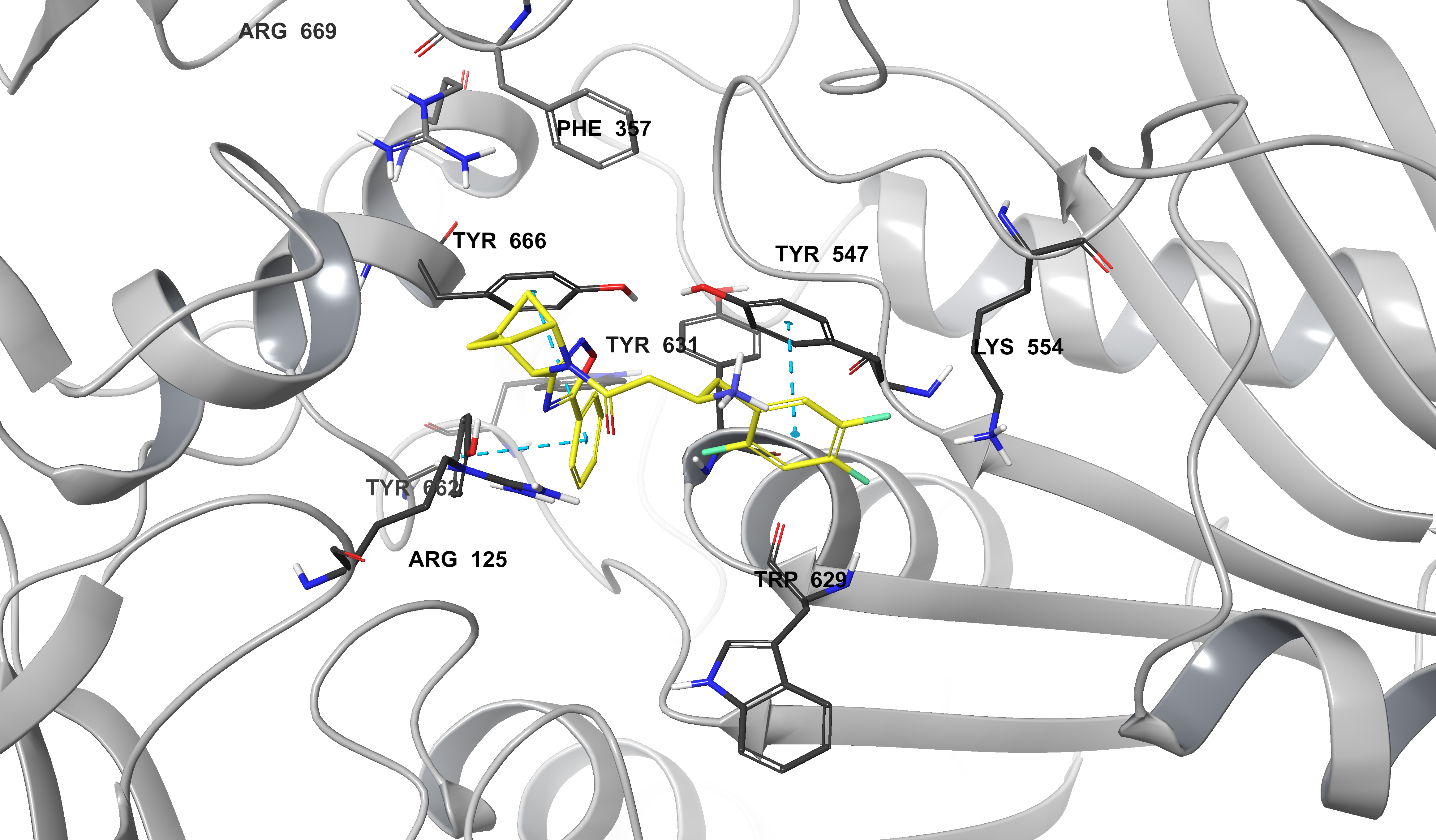

Supplement: Supplementary file 1 [file pharmaceuticals-18-00642-s001.zip › Docking images/Fig5-3a.png]

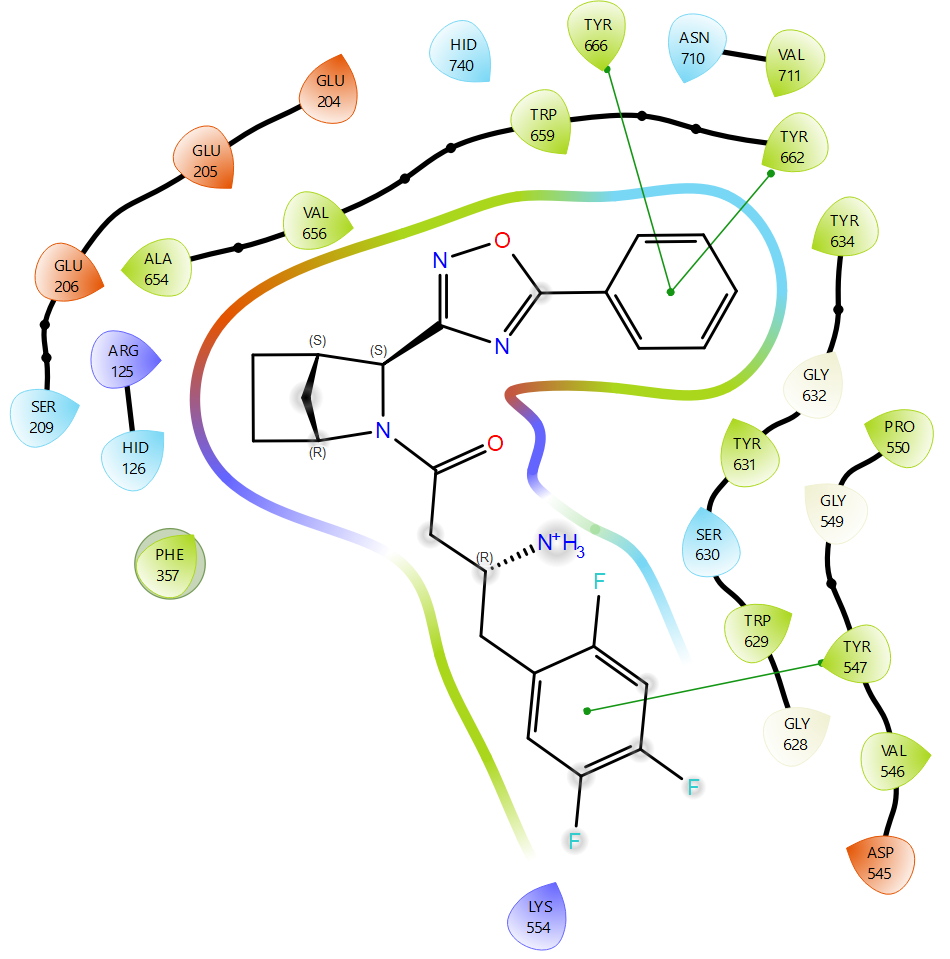

Supplement: Supplementary file 1 [file pharmaceuticals-18-00642-s001.zip › Docking images/Fig5-3a_LID.png]

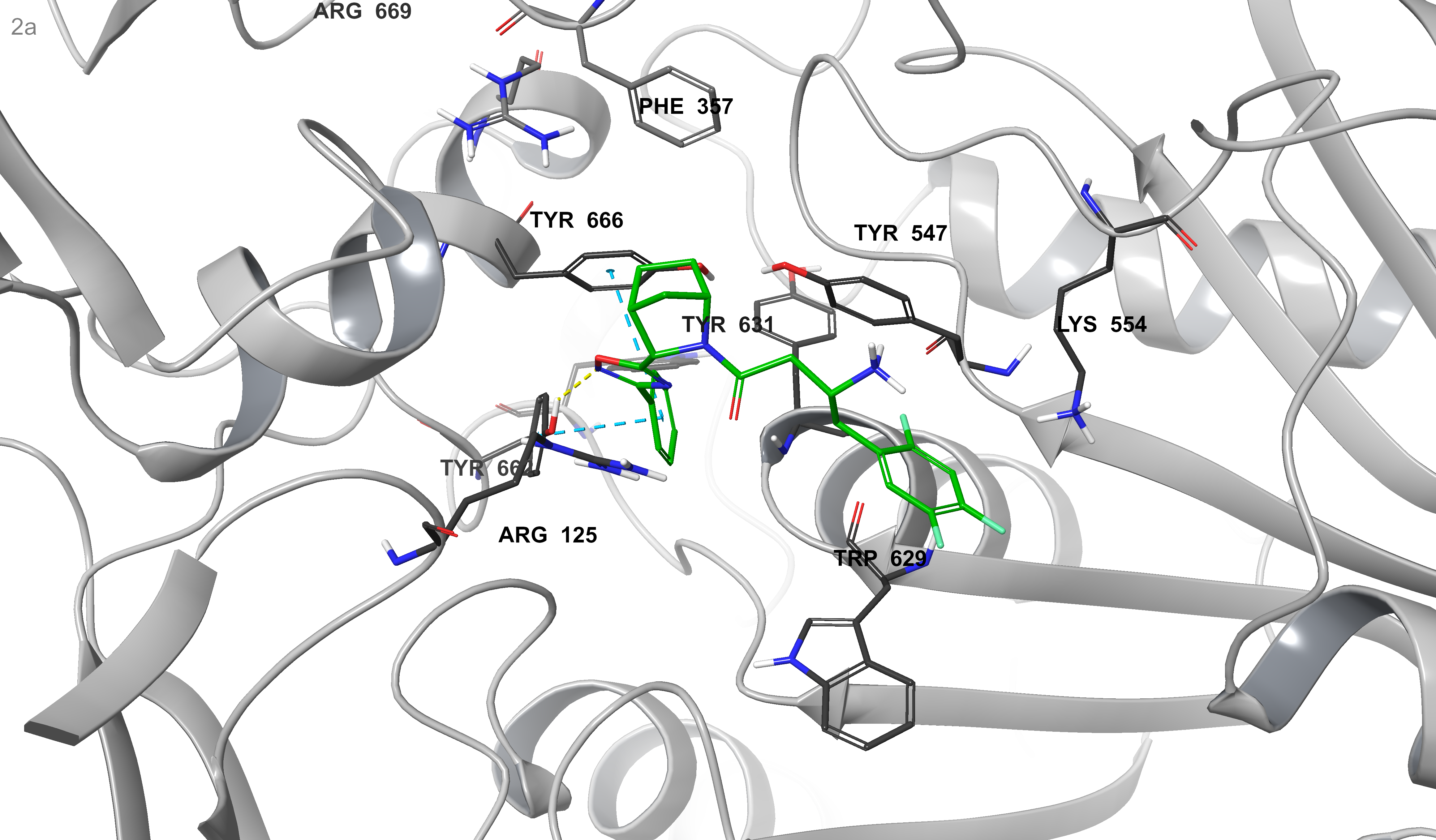

Supplement: Supplementary file 1 [file pharmaceuticals-18-00642-s001.zip › Docking images/Fig5-9a.png]

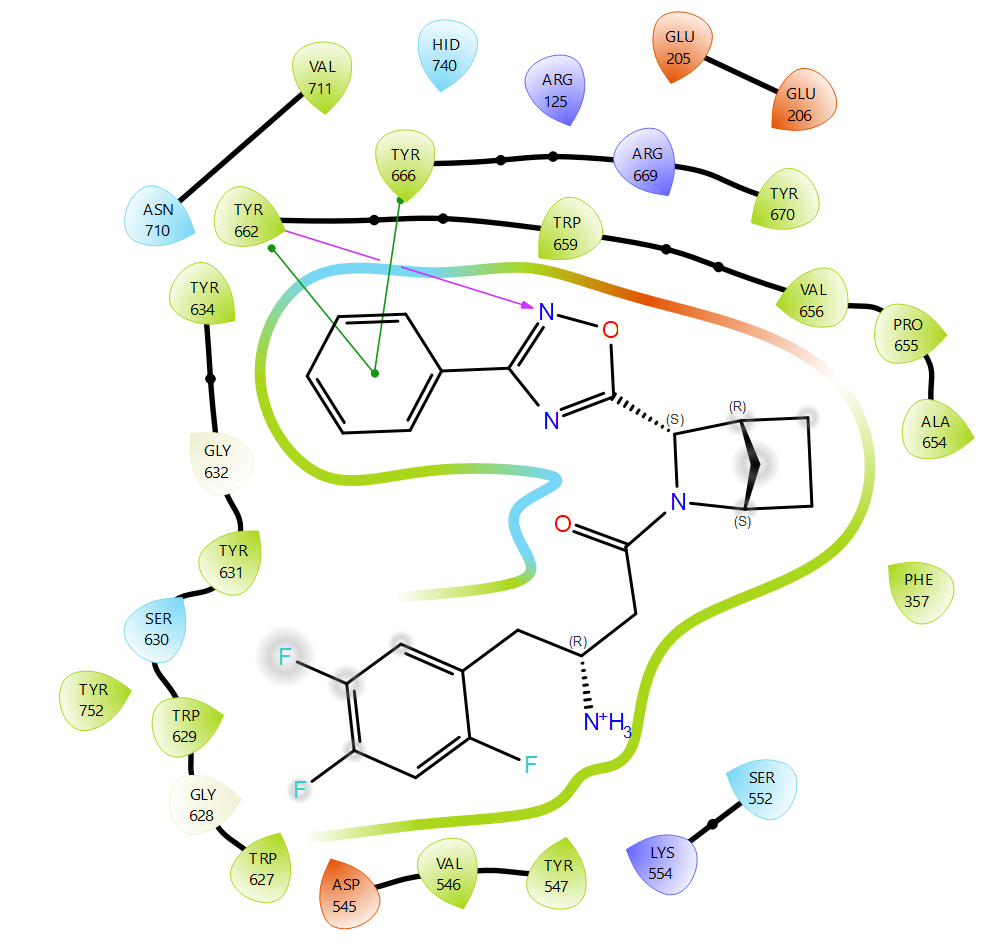

Supplement: Supplementary file 1 [file pharmaceuticals-18-00642-s001.zip › Docking images/Fig5-9aLID.png]

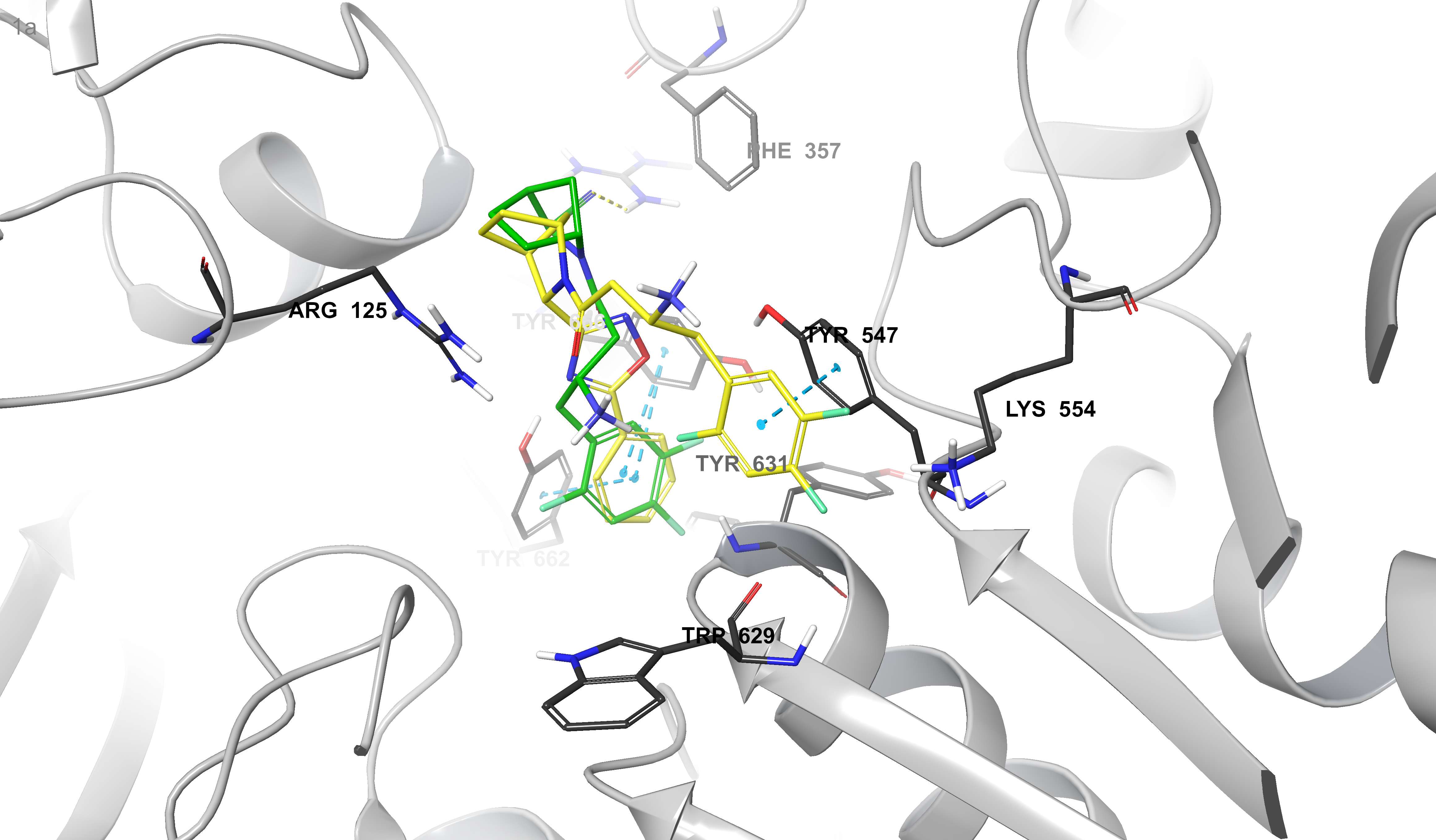

Supplement: Supplementary file 1 [file pharmaceuticals-18-00642-s001.zip › Docking images/SI_Neogliptin_3a.png]

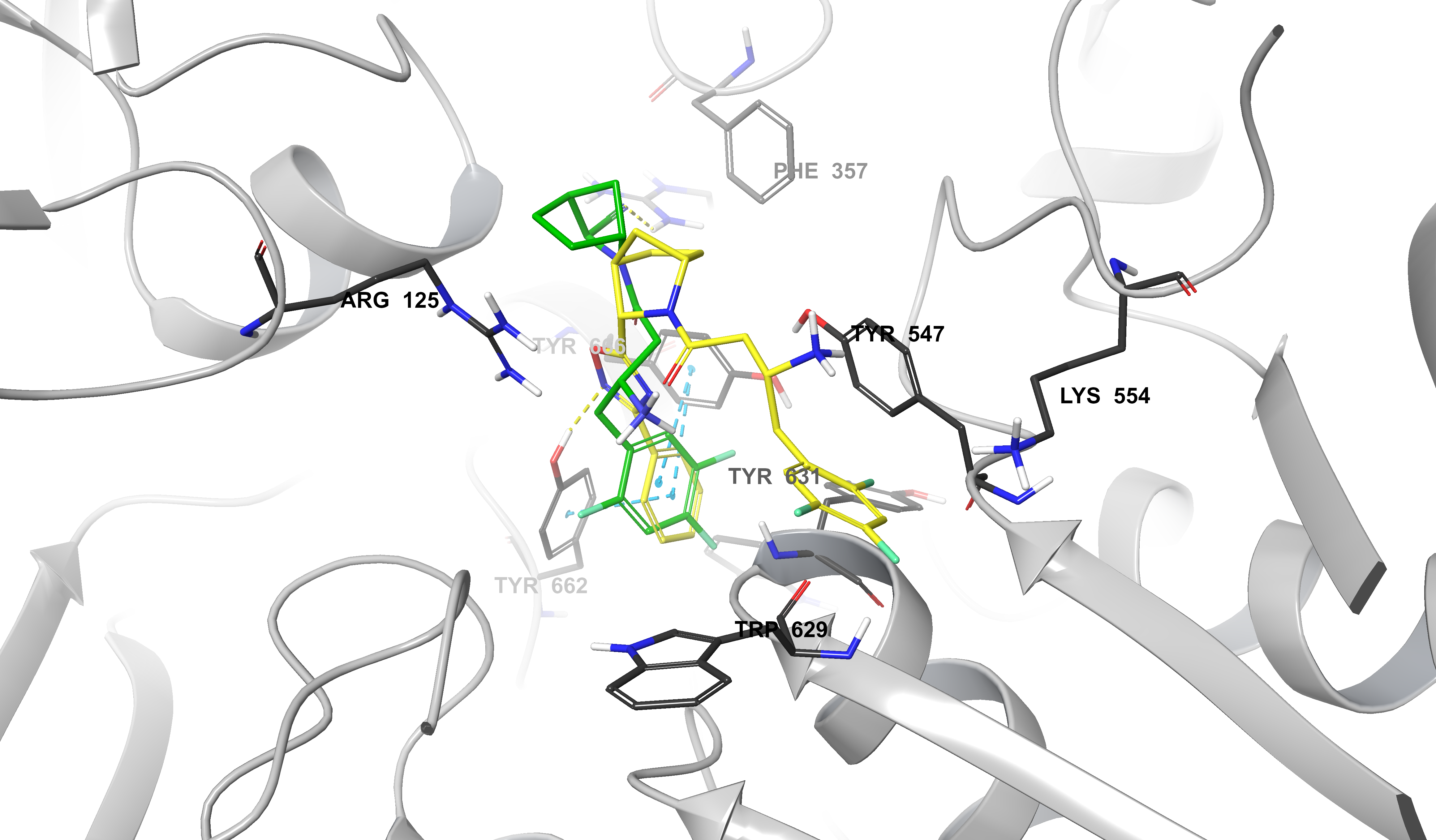

Supplement: Supplementary file 1 [file pharmaceuticals-18-00642-s001.zip › Docking images/SI_Neogliptin_9a.png]
