# Supplementary material for: 3-(3-Azabicyclo[2, 2, 1]heptan-2-yl)-1,2,4-oxadiazoles as Novel Potent DPP-4 Inhibitors to Treat T2DM
Source: Pharmaceuticals (Basel). 2025 Apr 28;18(5):642. doi: 10.3390/ph18050642 (PMC12114571; doi:10.3390/ph18050642)
Supplement: Supplementary file 1 [file 3b_COSY alifatic region.pdf › NMR/3b_NMR/3b_COSY alifatic region.pdf]

ULZ-555-1 DMSO-D6 Dsh 23_07_10-2/03 COSY

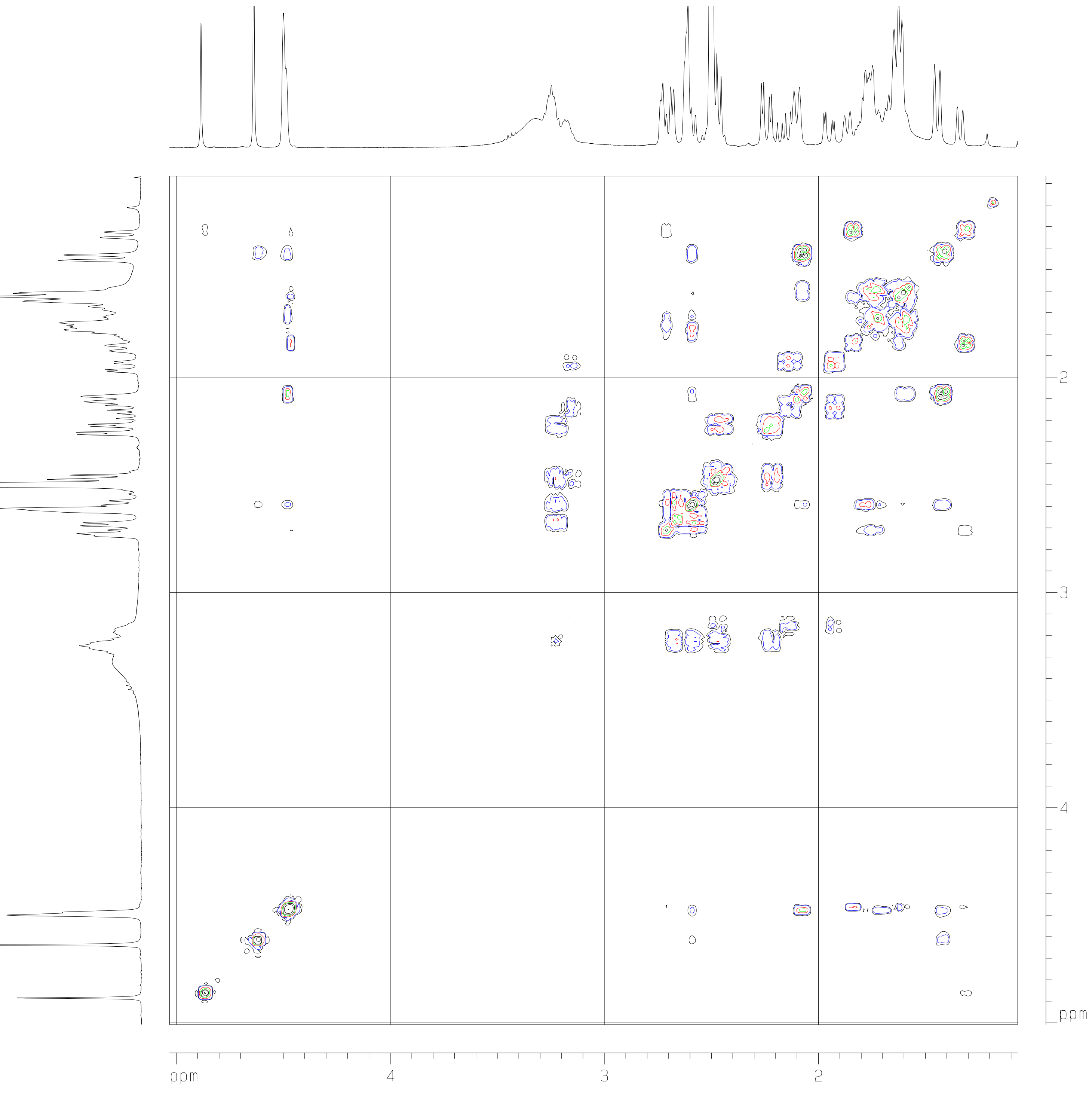

Current Data Parameters

NAME	ULZ-555-1
EXPNO	30
PROCNO	1

F2 - Acquisition Parameters

Date_	20230711
Time	12.07
INSTRUM	spect
PROBHD	5 mm Multinucl
PULPROG	cosygpqf
TD	1024
SOLVENT	DMSO
NS	1
DS	16
SWH	3930.818 Hz
FIDRES	3.838689 Hz
AQ	0.1303028 sec
RG	50
DW	127.200 usec
DE	6.00 usec
TE	0.0 K
d0	0.00000300 sec
D1	1.00000000 sec
d13	0.00000400 sec
D16	0.00010000 sec
IN0	0.00025501 sec
MCREST	0.00000000 sec
MCWRK	1.00000000 sec

======== CHANNEL f1 ========

NUC1	1H
P0	10.00 usec
P1	10.00 usec
PL1	0.00 dB
SFO1	400.1318590 MHz

====== GRADIENT CHANNEL =====

GPNAM1	SINE.100
GPNAM2	SINE.100
GPX1	0.00 %
GPX2	0.00 %
GPY1	0.00 %
GPY2	0.00 %
GPZ1	20.00 %
GPZ2	20.00 %
P16	1600.00 usec

F1 - Acquisition parameters

ND0	1
TD	512
SFO1	400.1319 MHz
FIDRES	7.658938 Hz
SW	9.800 ppm
FnMODE	QF

F2 - Processing parameters

SI	1024
SF	400.1300108 MHz
WDW	QSINE
SSB	0
LB	0.00 Hz
GB	0
PC	0.60

F1 - Processing parameters

SI	1024
MC2	QF
SF	400.1300120 MHz
WDW	QSINE
SSB	0
LB	0.00 Hz
GB	0

2D NMR plot parameters

CX2	15.00 cm
CX1	15.00 cm
F2PLO	5.032 ppm
F2LO	2013.28 Hz
F2PHI	1.069 ppm
F2HI	427.90 Hz
F1PLO	5.008 ppm
F1LO	2004.04 Hz
F1PHI	1.065 ppm
F1HI	426.30 Hz
F2PPMCM	0.26414 ppm/cm
F2HZCM	105.69191 Hz/cm
F1PPMCM	0.26287 ppm/cm
F1HZCM	105.18275 Hz/cm
