# Supplementary material for: 3-(3-Azabicyclo[2, 2, 1]heptan-2-yl)-1,2,4-oxadiazoles as Novel Potent DPP-4 Inhibitors to Treat T2DM
Source: Pharmaceuticals (Basel). 2025 Apr 28;18(5):642. doi: 10.3390/ph18050642 (PMC12114571; doi:10.3390/ph18050642)
Supplement: Supplementary file 1 [file pharmaceuticals-18-00642-s001.zip › NMR/6a,b_NMR/6a,b_COSY.pdf]

ULZ-525 DMSO-D6 Dsh 23_03_28-5/04 COSY

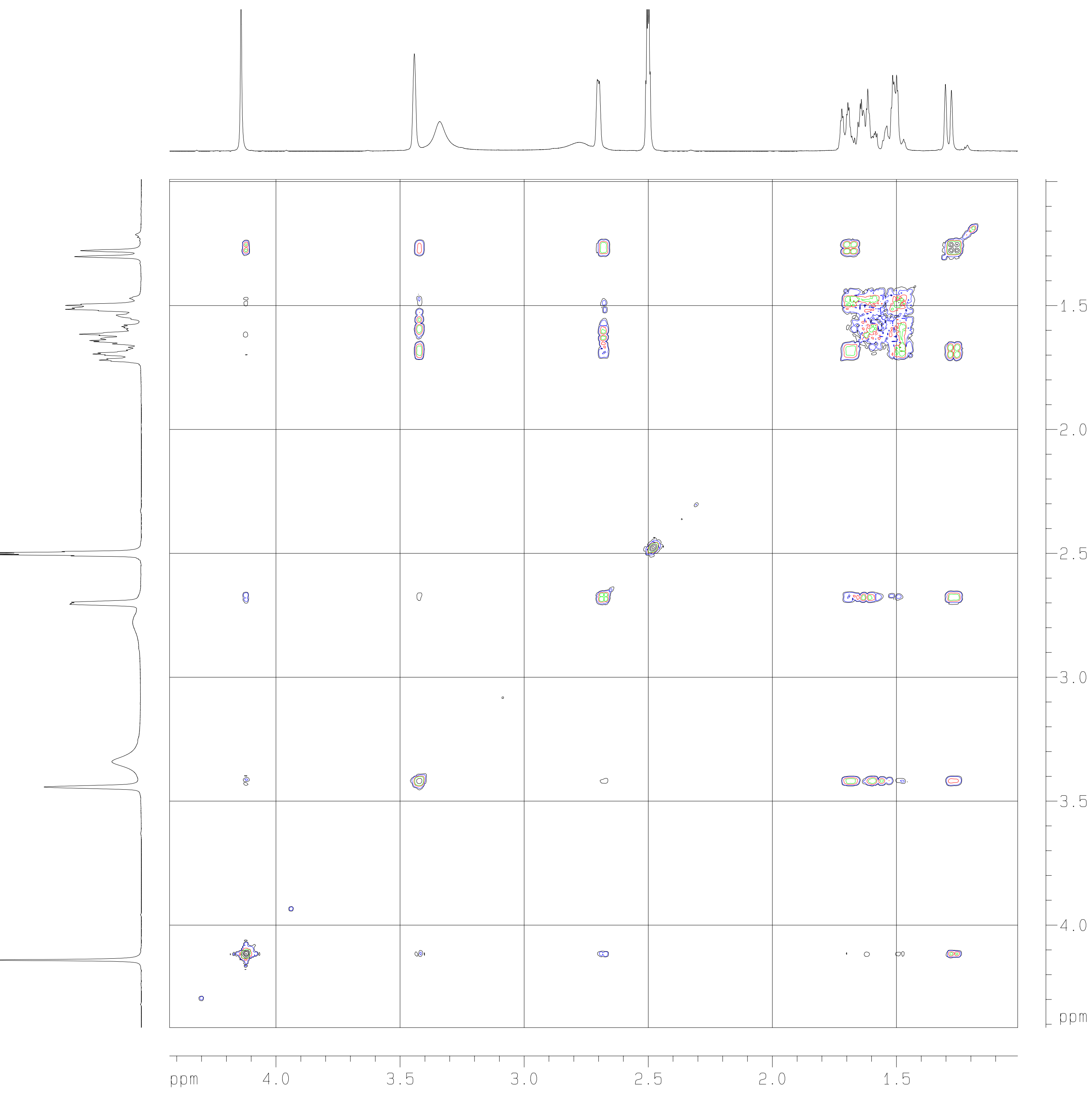

Current Data Parameters

NAME	ULZ-525
EXPNO	30
PROCNO	1

F2 - Acquisition Parameters

Date_	20230331
Time	12.58
INSTRUM	spect
PROBHD	5 mm Multinucl
PULPROG	cosygpqf
TD	1024
SOLVENT	DMSO
NS	1
DS	16
SWH	2003.205 Hz
FIDRES	1.956255 Hz
AQ	0.2556404 sec
RG	20
DW	249.600 usec
DE	6.00 usec
TE	0.0 K
d0	0.00000300 sec
D1	1.00000000 sec
d13	0.00000400 sec
D16	0.00010000 sec
IN0	0.00049984 sec
MCREST	0.00000000 sec
MCWRK	1.00000000 sec

======== CHANNEL f1 ========

NUC1	1H
P0	10.00 usec
P1	10.00 usec
PL1	0.00 dB
SFO1	400.1310003 MHz

====== GRADIENT CHANNEL =====

GPNAM1	SINE.100
GPNAM2	SINE.100
GPX1	0.00 %
GPX2	0.00 %
GPY1	0.00 %
GPY2	0.00 %
GPZ1	20.00 %
GPZ2	20.00 %
P16	1600.00 usec

F1 - Acquisition parameters

ND0	1
TD	512
SFO1	400.131 MHz
FIDRES	3.907520 Hz
SW	5.000 ppm
FnMODE	QF

F2 - Processing parameters

SI	1024
SF	400.1300108 MHz
WDW	QSINE
SSB	0
LB	0.00 Hz
GB	0
PC	0.60

F1 - Processing parameters

SI	1024
MC2	QF
SF	400.1300120 MHz
WDW	QSINE
SSB	0
LB	0.00 Hz
GB	0

2D NMR plot parameters

CX2	15.00 cm
CX1	15.00 cm
F2PLO	4.429 ppm
F2LO	1772.04 Hz
F2PHI	1.011 ppm
F2HI	404.62 Hz
F1PLO	4.413 ppm
F1LO	1765.95 Hz
F1PHI	0.991 ppm
F1HI	396.37 Hz
F2PPMCM	0.22783 ppm/cm
F2HZCM	91.16148 Hz/cm
F1PPMCM	0.22819 ppm/cm
F1HZCM	91.30571 Hz/cm
