# Supplementary material for: 3-(3-Azabicyclo[2, 2, 1]heptan-2-yl)-1,2,4-oxadiazoles as Novel Potent DPP-4 Inhibitors to Treat T2DM
Source: Pharmaceuticals (Basel). 2025 Apr 28;18(5):642. doi: 10.3390/ph18050642 (PMC12114571; doi:10.3390/ph18050642)
Supplement: Supplementary file 1 [file pharmaceuticals-18-00642-s001.zip › NMR/8b_NMR/8b_NMR 13C alifatic region.pdf]

ULZ-517 DMSO-D6 Dsh 22_11_16-4/14 NMR 13C

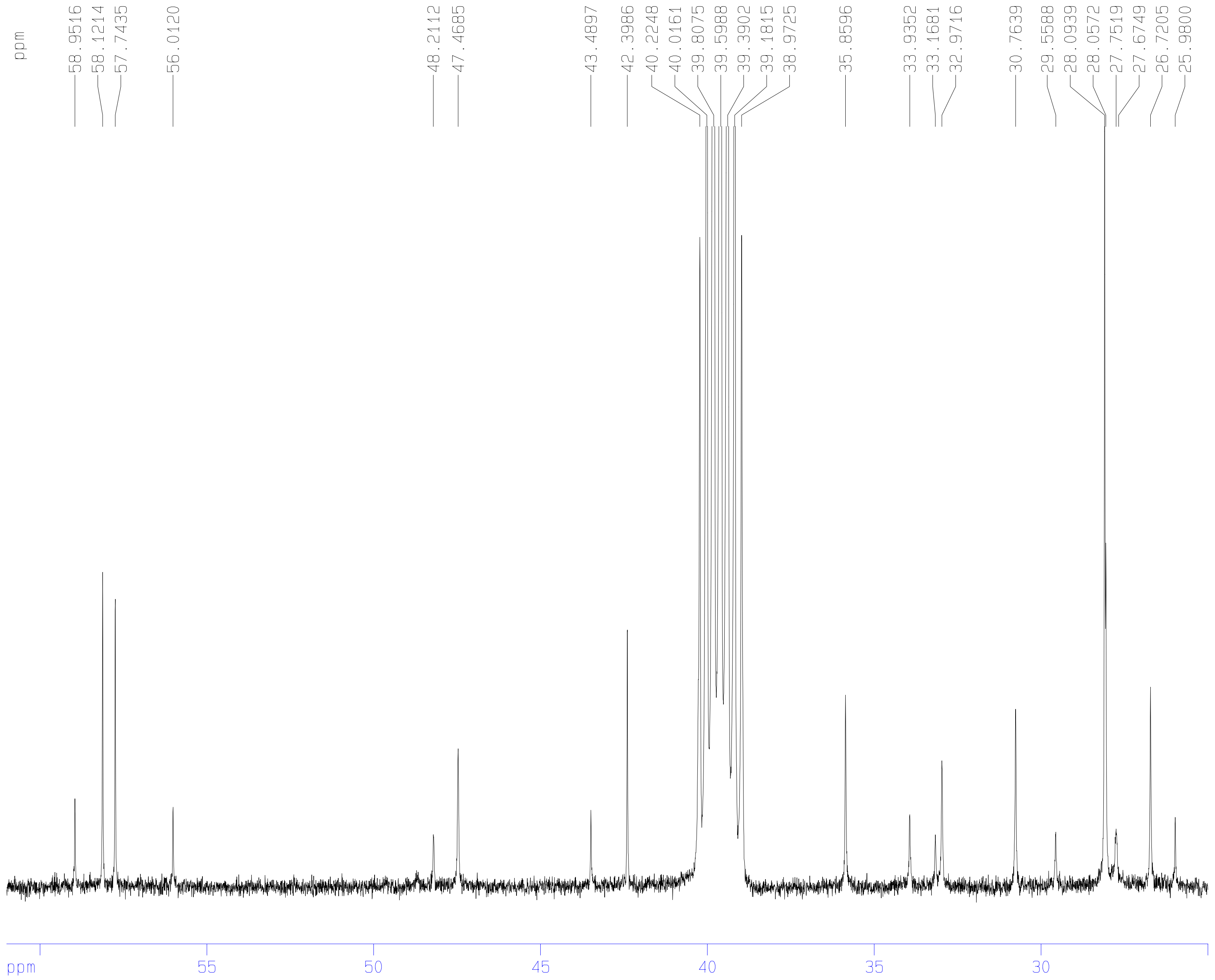

Current Data Parameters	
NAME	ulz-517
EXPNO	13
PROCNO	1
F2 - Acquisition Parameters	
Date_	20221122
Time	10.51
INSTRUM	spect
PROBHD	5 mm Multinucl
PULPROG	zgpg
TD	32768
SOLVENT	DMSO
NS	40306
DS	0
SWH	19083.969 Hz
FIDRES	0.582397 Hz
AQ	0.8585716 sec
RG	16384
DW	26.200 usec
DE	6.00 usec
TE	295.3 K
D1	1.00000000 sec
d11	0.03000000 sec
DELTA	0.89999998 sec
MCREST	0.00000000 sec
MCWRK	0.01500000 sec
======== CHANNEL f1 ========	
NUC1	13C
P1	7.00 usec
PL1	-3.00 dB
SFO1	100.6222266 MHz
======== CHANNEL f2 ========	
CPDPRG2	waltz16
NUC2	1H
PCPD2	100.00 usec
PL2	-2.00 dB
PL12	18.00 dB
PL13	18.00 dB
SFO2	400.1320000 MHz
F2 - Processing parameters	
SI	32768
SF	100.6128086 MHz
WDW	EM
SSB	0
LB	0.10 Hz
GB	0
PC	0.80
1D NMR plot parameters	
CX	21.00 cm
CY	80.00 cm
F1P	61.000 ppm
F1	6137.38 Hz
F2P	25.000 ppm
F2	2515.32 Hz
PPMCM	1.71429 ppm/cm
HZCM	172.47911 Hz/cm
