# Supplementary material for: 3-(3-Azabicyclo[2, 2, 1]heptan-2-yl)-1,2,4-oxadiazoles as Novel Potent DPP-4 Inhibitors to Treat T2DM
Source: Pharmaceuticals (Basel). 2025 Apr 28;18(5):642. doi: 10.3390/ph18050642 (PMC12114571; doi:10.3390/ph18050642)
Supplement: Supplementary file 1 [file pharmaceuticals-18-00642-s001.zip › NMR/8b_NMR/8b_NMR 13C aromatic region.pdf]

ULZ-517 DMSO-D6 Dsh 22_11_16-4/14 NMR 13C

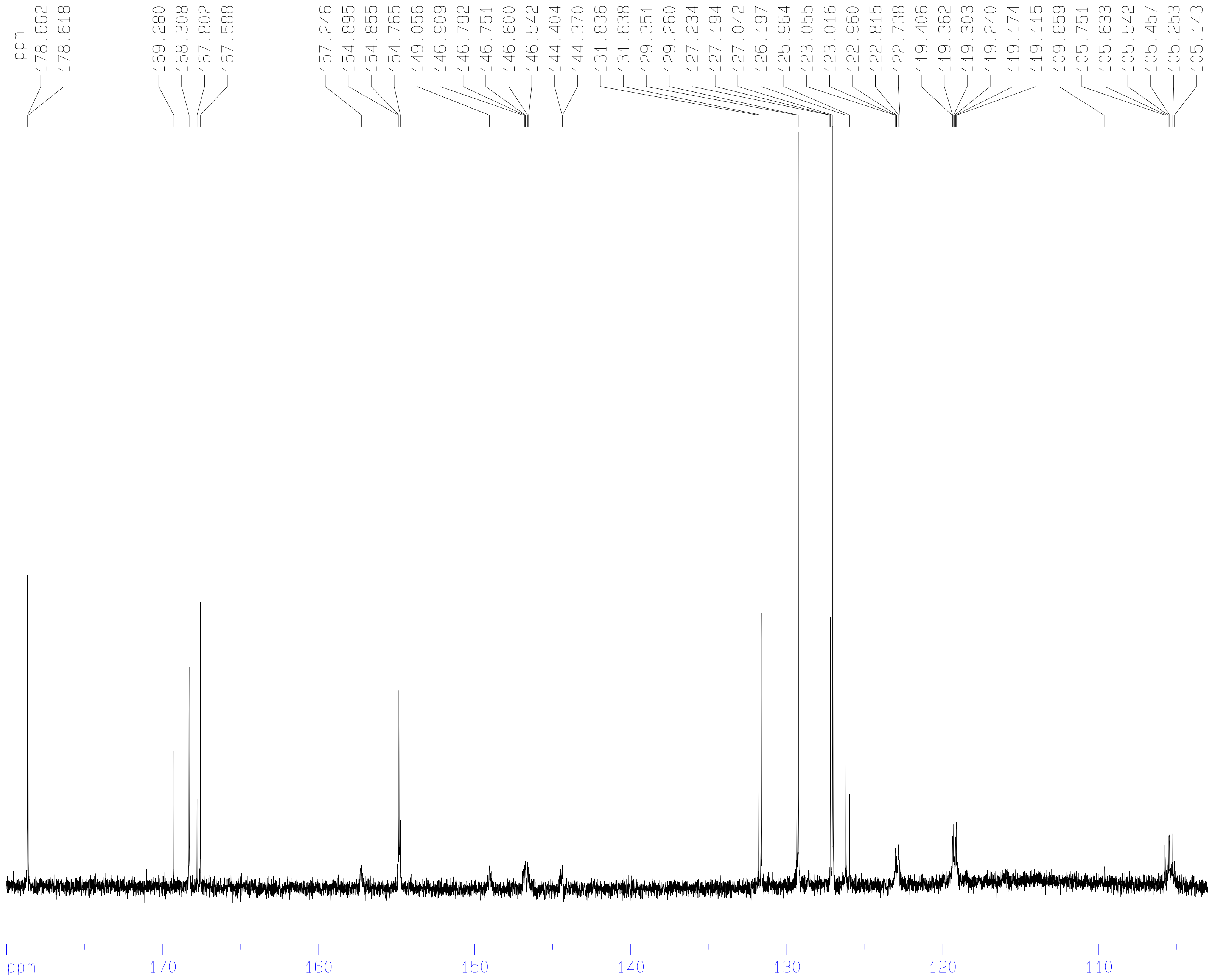

Current Data Parameters
NAME ulz-517
EXPNO 13
PROCNO 1

F2 - Acquisition Parameters
Date_ 20221122
Time 10.51
INSTRUM spect
PROBHD 5 mm Multinucl
PULPROG zgpg
TD 32768
SOLVENT DMSO
NS 40306
DS 0
SWH 19083.969 Hz
FIDRES 0.582397 Hz
AQ 0.8585716 sec
RG 16384
DW 26.200 usec
DE 6.00 usec
TE 295.3 K
D1 1.00000000 sec
d11 0.03000000 sec
DELTA 0.89999998 sec
MCREST 0.00000000 sec
MCWRK 0.01500000 sec

======== CHANNEL f1 ========
NUC1 13C
P1 7.00 usec
PL1 -3.00 dB
SFO1 100.6222266 MHz

======== CHANNEL f2 ========
CPDPRG2 waltz16
NUC2 1H
PCPD2 100.00 usec
PL2 -2.00 dB
PL12 18.00 dB
PL13 18.00 dB
SFO2 400.1320000 MHz

F2 - Processing parameters
SI 32768
SF 100.6128086 MHz
WDW EM
SSB 0
LB 0.10 Hz
GB 0
PC 0.80

1D NMR plot parameters
CX 21.00 cm
CY 80.00 cm
F1P 180.000 ppm
F1 18110.30 Hz
F2P 103.000 ppm
F2 10363.12 Hz
PPMCM 3.66667 ppm/cm
HZCM 368.91364 Hz/cm
